# Soft Micromanipulation Robot for Real‐Time Adaptive Multimodal Operation

**DOI:** 10.1002/advs.202515784

**Published:** 2025-10-21

**Authors:** Zhuowei Li, Xiaotian Lin, Zhoujie Zhu, Yibo Zhu, Yanping Zhou, Jing Li, Chris Gerada, He Zhang, Songlin Zhuang

**Affiliations:** ^1^ Yongjiang Laboratory Ningbo Zhejiang 315201 China; ^2^ University of Nottingham Ningbo China Ningbo 315100 China; ^3^ Power Electronics, Machines and Control Research Group University of Nottingham Nottingham NG8 1BB UK

**Keywords:** bio‐inspired design, biomedical engineering, micromanipulation, microsurgery, soft robotic

## Abstract

Micromanipulation robots hold immense promise for biomedical applications, yet they remain fundamentally limited by three persistent challenges: cross‐scale target heterogeneity, spatially constrained workspaces, and integrated multimodal operation requirements. Here, a soft micromanipulation robot (SMR) capable of omnidirectional, micrometer‐precision manipulation via a hollow multi‐notch agonist‐antagonist mechanism is presented. Combining ± 180° bending and 360° rotation for full‐angle operation, this bio‐inspired design achieves 14 µm positioning accuracy, enabling reliable handling of single‐cell‐sized objects. The SMR adapts in situ to sensitive biosamples and limited workspaces, supporting diverse manipulation modes including aspiration, transfer, programmable assembly, targeted microinjection, and localized cutting of biospecimens. To evaluate biomedical applicability, an assembly experiment with human kidney cell spheres, which is essential for establishing co‐culture models in new drug development is designed. The SMR successfully aspirated, transferred, and precisely positioned multiple assembloids onto ring‐shaped biochips, achieving programmable assembly within limited workspaces. The SMR has the potential to be a flexible and adaptable platform for performing delicate operations in various biomedical scenarios, such as in vitro modeling, drug testing, and microscale surgery.

## Graphical abstract

This manuscript proposes a soft micromanipulator robot (SMR) for “full‐angle” precision biomedical operations. Bio‐inspired SMR achieves omnidirectional motion, micrometer precision, and multimodal capabilities including aspiration, transfer, rotation, assembly, injection, and cutting. It adapts to perform diverse operations on biological targets across scales within limited spaces, enabling extensive possibilities for in vitro modeling, drug testing, and microscale surgery applications.

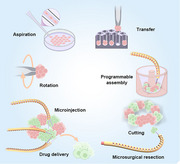

## 1 Introduction

**Figure 1:**
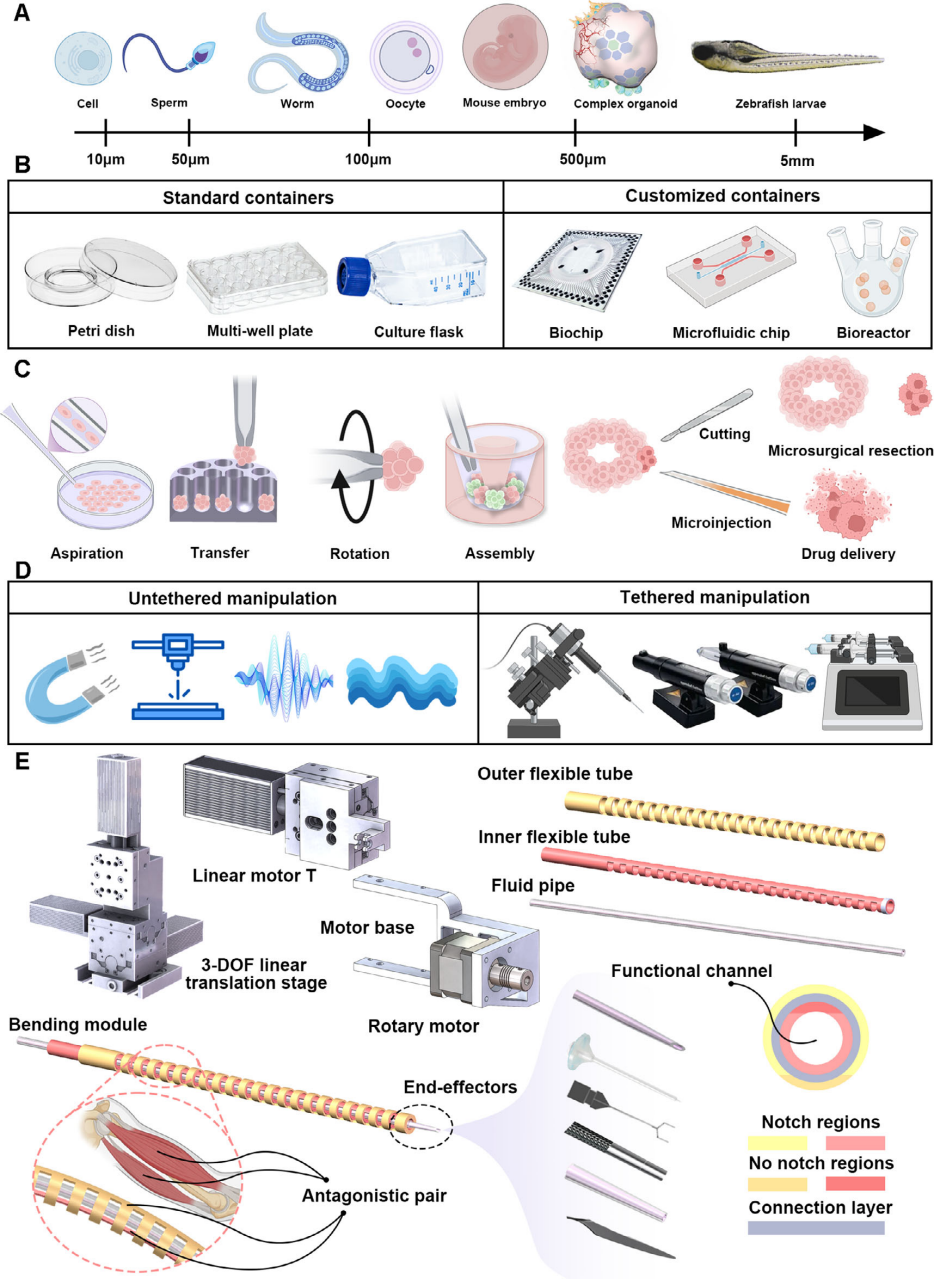
Three fundamental challenges in biological micromanipulation and the soft micromanipulation robot. A) Hierarchical biological targets spanning sub‐100 µm (cells, sperm), 100–500 µm organisms (worms, oocytes, embryos, complex organoids), and mm‐scale (zebrafish larvae). B) Workspace constraints imposed by standard containers (Petri dishes, multiwell plates, culture flasks) and customized containers (biochips, microfluidic chips, bioreactors). C) Core manipulation requirements defined by six essential tasks: aspiration, transfer, rotation, assembly, cutting, and microinjection. D) The operational methods are categorized as untethered (magnetic, optical, acoustic, and fluid fields) and tethered (robotic manipulators with end‐effectors). E) Soft micromanipulation robot: Schematic of the proposed system integrating a 3‐DOF motion platform, 360° rotary actuation, and ± 180° bending module for comprehensive biological manipulation. Its geometric adaptation and micron‐scale precision overcome scale, workspace, and dexterity challenges.

The revelation of the microscopic world, unveiled by the invention of the microscope, has fundamentally transformed our understanding of life and disease.^[^ ^1^, ^2^ ^]^ Many physiological and pathological processes, ranging from cellular metabolism to the onset of cancer, are now known to originate at the micron scale.^[^ ^3^, ^4^ ^]^ This insight has inspired researchers to harness advances in engineering, robotics, and control theory to develop micromanipulators capable of replicating macro‐scale manipulation skills within the micro‐world, effectively realizing a “fantastic voyage” into living systems. Since the development of the first micromanipulator in the 1990s, these technologies have been increasingly adopted in developmental biology, disease diagnosis, and personalized medicine, accelerating progress across diverse biomedical domains.^[^ ^5^, ^6^ ^]^ Despite promising progress, three key scientific challenges still constrain further advancement in the field of micromanipulation.^[^ ^7^, ^8^, ^9^, ^10^, ^11^ ^]^ Biological targets span orders of magnitude in size, from cells to millimeter‐scale model organisms, each exhibiting distinct morphological complexity and thus demanding manipulators that combine micron‐level accuracy with adaptive mobility and multimodal functionality (**Figure** **1**A). These operations are typically performed within spatially constrained workspaces such as biochips or multiwell plates, where physical boundaries severely limit access and dexterity, posing fundamental questions about how to maintain precision while enabling reconfigurability (Figure 1B). Furthermore, the diversity of required manipulations, including aspiration, transfer, rotation, cutting, injection, and assembly, necessitates a unified platform capable of integrating multiple manipulation modalities with coordinated execution (Figure 1C). Addressing the intertwined challenges posed by cross‐scale heterogeneity, restricted workspace accessibility, and multimodal integration is essential for developing versatile micromanipulators.

In response to these challenges, both non‐contact and tethered manipulation technologies have been developed, each offering unique capabilities (Figure 1D). Contact‐free micromanipulation strategies have been developed, leveraging field‐induced forces (magnetic,^[^ ^12^, ^13^, ^14^ ^]^ optical,^[^ ^15^, ^16^, ^17^ ^]^ acoustic,^[^ ^18^, ^19^, ^20^ ^]^ microfluidic^[^ ^21^, ^22^, ^23^ ^]^) for 3D manipulation. Their non‐contact nature enables gentle handling across scales, capturing 1‐µm bacteria via optical traps,^[^ ^24^, ^25^ ^]^ transporting submillimeter scale organoids through acoustic streaming,^[^ ^18^, ^26^, ^27^ ^]^ and rotating mm‐scale zebrafish larvae using magnetic fields,^[^ ^28^, ^29^ ^]^ significantly reducing mechanical damage to biological samples. Each modality offers distinct advantages for specific scenarios. Magnetic manipulation enables multimodal control of labeled targets across scales, facilitating precise rotation, translation, and coordinated control of biological specimens.^[^ ^30^, ^31^, ^32^ ^]^ Optical tweezers achieve sub‐micron positioning accuracy, ideal for single‐cell manipulation.^[^ ^33^, ^34^, ^35^ ^]^ Acoustic fields provide label‐free patterning capabilities for biological specimens such as embryos and multicellular clusters.^[^ ^20^, ^27^, ^36^ ^]^ Microfluidic systems enable controlled transport through precisely engineered channels.^[^ ^37^, ^38^, ^39^ ^]^ Although these untethered methods minimize mechanical disturbance, their inherent limitations in force generation and their restricted DOFs render them unsuitable for contact‐dependent operations such as aspiration, microinjection, and microdissection, which require precise mechanical interaction with biological samples. Thus, there is a great demand to develop manipulators with unified cross‐scale precision, spatial adaptability, and multimodal force modulation.

Faced with the force limitations of untethered methods, research has refocused on tethered approaches. Conventional micromanipulation platforms have sought enhanced dexterity through additional DOFs, demonstrating improved outcomes in dedicated applications.^[^ ^40^, ^41^, ^42^ ^]^ For instance, integrated rotational DOFs enable adaptive end‐effector realignment to arbitrary orientations, increasing sperm aspiration efficiency through precise angular control.^[^ ^43^, ^44^ ^]^ Similarly, adding rotational and thrusting DOFs optimizes puncture trajectories, reducing required puncture force and minimizing deformation during zebrafishes and oocytes microinjection.^[^ ^45^, ^46^, ^47^ ^]^ While these task‐specific innovations advance capabilities for targeted scenarios like sperm capture and embryo manipulation, they remain confined to application‐specific configurations. These specialized innovations lack a unified architecture capable of concurrently addressing biological diversity, geometric restrictions, and combinatorial manipulation requirements.

To address these challenges, soft continuum robots inspired by biological appendages (e.g., elephant trunks and octopus arms) offer a compelling alternative by combining intrinsic compliance, continuous deformability, and high dexterity for safe interaction with delicate samples.^[^ ^48^, ^49^, ^50^ ^]^ Their proven success in macro‐scale robotic surgery demonstrates their ability to navigate narrow natural lumens to perform complex tasks including tumor biopsy and tissue resection.^[^ ^51^, ^52^, ^53^, ^54^ ^]^ This makes them conceptually well‐suited for micromanipulation tasks in spatially restricted workspaces such as biochips and multiwell plates. However, current designs are primarily tailored to surgical applications, where sub‐millimeter precision is generally acceptable and operational demands differ significantly from those at the microscale. Furthermore, they lack the cross‐scale adaptability, micron‐level accuracy, and multimodal functionality required to handle targets ranging from single cells to millimeter‐scale organisms, or to integrate diverse operations such as aspiration, rotation, injection, and assembly under microscopic conditions.

This paper presents a soft micromanipulation robot (SMR) that integrates an asymmetrically notched structure with antagonistic actuation for large range bending (± 180°), along with modular actuators enabling continuous rotation (360°) and 3‐DOF translation. This design enables, for the first time, “full‐angle” operation under a microscope with micron‐level accuracy (± 14 µm). Stable curvature control is achieved by antagonistic actuation, supported by a kinematic model that predicts the end‐effectors position and orientation. A rapid calibration algorithm is proposed to accurately and automatically perform 3D coordinate transformations, facilitating real‐time compensation for end‐effector variations and task‐specific adjustments. The SMR achieves a 3.4 ×– 6.8 × increase in operational range compared to conventional rigid manipulators. The SMR performed multifunctional operations—including aspiration, transfer, programmable assembly, microinjection, and cutting—on diverse targets ranging from fluorescent microbeads to human embryonic kidney 293 (293T) cell clusters. Specifically, we deployed the SMR to programmatically assemble mCherry/GFP‐labeled cell clusters into ring‐shaped biostructures within confined spaces, establishing viable platforms for drug screening. Furthermore, we fabricated tumor‐mimetic architectures on biochips and performed precision interventions, including targeted resection and localized drug delivery, without damaging the surrounding microenvironment.

## 2 Results and Discussion

### 2.1 Design of a Soft Micromanipulation Robot

The soft micromanipulation robot (SMR) (Figure 1E) integrates three functionally complementary modules–bending, rotation, and translation–to address cross‐scale biological heterogeneity, workspace constraints, and multimodal manipulation requirements. The SMR comprises a 3‐DOF linear translation stage (MX7600, Siskiyou) supporting a linear motor T, that defines the initial orientation angle of the end‐effector. A rotary motor (ST‐35, UMot) is mounted on the guide rails of Motor T and is connected to the dual‐tube bending module. This core component features coaxially nested inner (agonist, red) and outer (antagonist, yellow) tubes fused distally.

This dual‐tube structure acts as a bionic agonist‐antagonist pair. During actuation, the linear motor T displaces the inner tube (agonist) linearly, transmitting tensile or compressive forces.^[^ ^55^, ^56^ ^]^ Tension shortens the effective length of the agonist relative to the antagonist tube, inducing bending, while the antagonist provides restorative force through inherent material elasticity. This dynamic equilibrium enables stable curvature control (± 180°), with asymmetric notches along the length of the tube programmably regulating bending radius (detailed in Section 2.2). Crucially, the required geometric symmetry for torsion‐free motion cannot be achieved through manual assembly. Thus, the structure is monolithically fabricated via photopolymerization, eliminating twist‐induced positioning errors critical for micron‐level precision.

A functional channel traversing the bending module accommodates various tethered microtools (micropipettes, microinjection needles, microelectrodes, etc.). Simultaneously, the rotary motor drives the entire bending module through continuous 360° rotation, reorienting end‐effectors to arbitrary spatial angles. The 3‐DOF translation stage complements these motions by dynamically compensating for end‐effector displacement during bending or rotation, thus keeping the target within the operational workspace. This coordinated motion control enables “full‐angle” manipulation (± 180° bend and 360° rotation) in confined environments such as multiwell plates and biochips. The SMR is remotely operated via a graphical interface, coordinating all modules in real time. This integrated design enables the SMR to perform six core micromanipulation tasks (aspiration, transfer, rotation, bioassembly, precision cutting, and targeted microinjection) across biological scales, ranging from single cells to millimeter‐scale specimens, supporting a broad range of complex operations in limited‐access workspaces.

### 2.2 Dual‐Model Control of Soft Micromanipulation Robots

**Figure 2:**
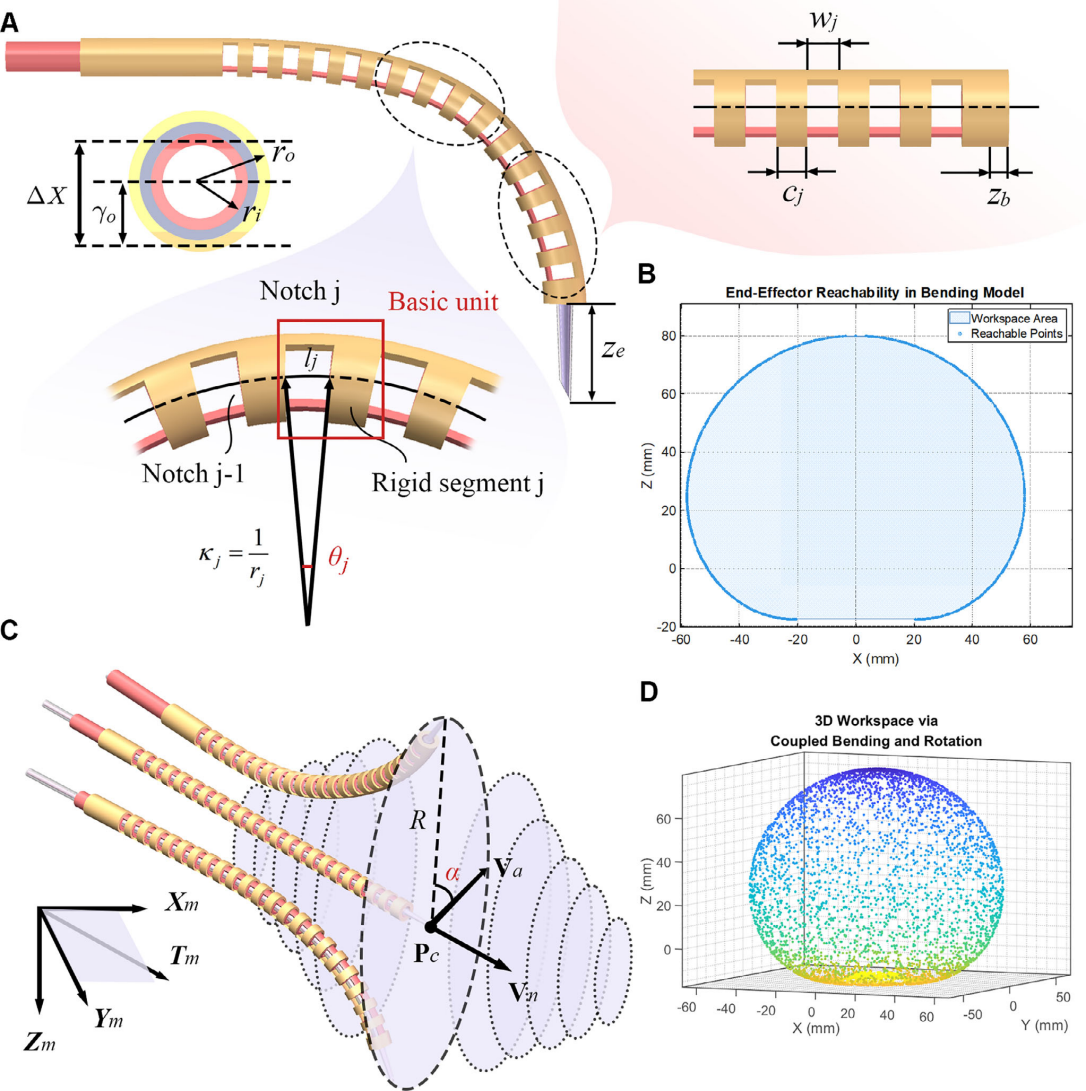
Dual‐mode kinematic modeling and workspace characterization of the SMR. A) Geometric parameterization of the SMR design, illustrating key parameters and variables in the bending model. B) Planar workspace of the bending model. End‐effector trajectories are predicted based on feed distance *d* of linear motor T, enabling real‐time trajectory tracking and angular adjustment under microscopy. C) Relationship between the SMR coordinate system and its rotational trajectory in 3D space. Different displacements *d* form different bending radii and different sizes of rotational circles. D) Full 3D reachable workspace generated by coordinated bending and rotation, illustrating omnidirectional access within a confined spherical volume.

To ensure the SMR remains within the microscope's field of view (FOV) during continuous angular adjustments, we developed a dual‐model coordinated control system comprising a bending model and a rotation model. The bending model determines the end‐effector's 3D pose (position and bending angle) from the displacement of the linear motor T, enabling large‐range directional deflection. Concurrently, the rotation model drives an independent rotary motor to achieve omnidirectional in‐plane angular adjustment, allowing reorientation without lateral drift (**Figure** **2**).

The bending kinematics of the SMR was modeled using a variable curvature method (Note S1, Supporting Information), accounting for the variable notch length *w*_*j*_ and rigid segment length *c*_*j*_ in each section *j* of the inner and outer tubes. Each notch and its adjacent rigid segment constitute a basic unit. When linear motor T applies a pull force to the inner tube, the asymmetrical notch design creates a moment arm between the neutral axes of the inner and outer tubes. This moment arm generates a bending torque, pulling the distal end of the outer tube toward the notched side (Figure S4, Supporting Information). Conversely, applying compressive force (e.g., pushing the inner tube) reverses the moment arm, inducing bending in the opposite direction. The bending model quantitatively relates the motor displacement *d* to the geometric tube parameters:

(1) $$d=\sum_{j=1}^{n}\frac{\Delta X_{j}w_{j}\theta_{j}}{l_{j}+\gamma_{o,j}\theta_{j}}$$

where Δ*X*_*j*_ denotes the distance between the neutral axes of inner and outer tubes for unit *j*, *w*_*j*_ represents the notch length of unit *j*, and θ_*j*_ is the bending angle at unit *j* that serves as the key output for orientation control. *l*_*j*_ is the centerline length of notched segment in unit *j*, and γ_*o*, *j*_ refers to the offset from outer tube's neutral axis to SMR neutral line.

The overall transformation from the base (first notch) to the end‐effector position is given by the homogeneous transformation matrix:

(2) $$T_{\text{base}}^{\text{ee}}=\left(\prod_{j=1}^{n}T_{\text{notch},j}T_{\text{rigid},j}\right)T_{z_{b}}T_{z_{e}}$$

where *T*_notch, *j*_ represents the transformation matrix for the *j*th notched segment, determined by its curvature (κ_*j*_) and centerline length (*l*_*j*_). *T*_rigid, *j*_ is the transformation matrix for the *j*th rigid segment. $T_{z_{b}}$ denotes the transformation accounting for the connection layer length between inner and outer tubes, and $T_{z_{e}}$ is the transformation accounting for the actual end‐effector length.

This model enables prediction of the end‐effector pose based on motor displacement *d* and tube geometry. The bending characteristics are determined by key geometric parameters, which can be adjusted to achieve desired performance, including notch length (*w*), rigid segment length (*c*), tube radii (*r*_*o*_, *r*_*i*_), and the number of units (*n*). When both notch length and rigid segment length are uniform across all units, the model simplifies to a constant curvature method. In this case, the bending occurs along a circular arc as demonstrated in Figure 2B, which illustrates the SMR's deformation profile using the bending model without rotational actuation.

The rotation model serves as the core module for dynamic angular adjustment of the end‐effector about the *T*_*m*_ axis (Figure 2C). It defines the end‐effector position (**P**_*e*_) during rotational motion:

(3) $$P_{e}=u_{R(d)}\sin\left(\alpha \right)\left(V_{n}\times V_{a}\right)+u_{R(\theta )}\cos\left(\alpha \right)V_{a}+P_{c}+u_{xyz}$$

where α denotes the rotation angle, which is actuated by the rotary motor. **u**_*R*(*d*)_ represents the rotation radius, whose magnitude is determined by the displacement *d* of the linear motor T in the bending model. **V**_*n*_ is the unit nominal vector parallel to the rotation axis *T*_*m*_, and **V**_*a*_ is an arbitrary unit vector lying in the rotation plane and orthogonal to **V**_*n*_. **P**_*c*_ is the position of the circle center in the world coordinate system. **u**_*xyz*_ accounts for the translational displacements along the *X*_*m*_, *Y*_*m*_ and *Z*_*m*_ axes, which are generated by the 3‐DOF linear translation stage.

Utilizing the bending model (Equations 1 and 2) and rotation model (Equation 3), we implement a visual closed‐loop control strategy that ensures operational safety and precision through three coordinated mechanisms. Collision avoidance is achieved by synergistically actuating the rotary motor and linear motor T, which dynamically adjusts the end‐effector trajectory to prevent contact with culture vessel boundaries and delicate biological specimens. Adaptive angle optimization enables real‐time adjustment of the operating angle α based on biospecimen morphology, establishing an optimal posture that maximizes efficiency while minimizing mechanically induced damage. Furthermore, vision servoing leverages continuous feedback of the rotation model parameters (rotation angle α and bending radius *R*_θ_), to drive micron‐level compensation (**u**_*xyz*_) via the 3‐DOF linear stage. This active correction maintains the end‐effector tip within ± 5.8 µm of the optical field center (Movie S1, Supporting Information), effectively compensating for modeling inaccuracies and overcoming FOV limitations.

This dual‐model cooperative framework establishes the foundation for precise SMR motion control. The bending model provides accurate prediction and control of the end‐effector's 3D position and orientation. The rotation model enables dynamic optimization of viewing and operational angles within the constrained workspace. Integrated active compensation (**u**_*xyz*_) effectively eliminates positioning errors induced by bending as well as rotation, and mitigates operational constraints arising from optical obstruction. Crucially, this decoupled kinematic framework enables comprehensive workspace characterization, as demonstrated by the 3D reachable point distribution in Figure 2D. The decoupled framework further supports implementation of advanced feedback controllers (e.g., PID, adaptive control,^[^ ^43^ ^]^ sliding mode control^[^ ^44^ ^]^) to minimize end‐effector positioning error. This significantly enhances the SMR's applicability in complex task micromanipulation scenarios.

### 2.3 Multi‐Angle Calibration and Accuracy Evaluation

The multi‐angle operation capability of the SMR arises from its additional bending and rotational DOF, but this flexibility introduces substantial calibration challenges. Conventional point‐to‐point calibration methods demand extensive manual intervention and are time‐consuming, limiting their applicability in time‐sensitive biological procedures that require complex manipulations, such as live cell assembly and high‐throughput screening. This creates an urgent need for efficient calibration methods tailored to robots with bending and rotating characteristics.

We define the SMR coordinate frame as *O*_*m*_ − *X*_*m*_ *Y*_*m*_ *Z*_*m*_ and the end‐effector frame as *O*_*e*_ − *X*_*e*_ *Y*_*e*_ *Z*_*e*_, with tip positions [*X*_*e*_, *Y*_*e*_, *Z*_*e*_]^*T*^ derived from dual kinematic models (Equations (2), (3)). The camera coordinate frame *O*_*c*_ − *X*_*c*_ *Y*_*c*_ *Z*_*c*_ relates to image coordinates *O*_*i*_ − *UV* through a known intrinsic matrix **K**. Our calibration objective is to determine the rigid transformation (rotation **R** and translation **t**) mapping end‐effector positions to camera coordinates as expressed in the projection equation:

(4) $$\left[\begin{array}{c} u\\ v \end{array}\right]=\frac{1}{Z_{c}}\underset{K}{\underset{︸}{\left[\begin{array}{ccc} f_{x} & 0 & c_{x}\\ 0 & f_{y} & c_{y} \end{array}\right]}}\cdot \left(R\cdot \left[\begin{array}{c} X_{e}\\ Y_{e}\\ Z_{e} \end{array}\right]+t\right)$$

where [*u*, *v*]^*T*^ are pixel coordinates in *O*_*i*_ and [*X*_*e*_, *Y*_*e*_, *Z*_*e*_] are coordinates in *O*_*e*_. **K** is a camera intrinsic matrix contains the focal lengths (*f*_*x*_, *f*_*y*_) and principal points (*c*_*x*_, *c*_*y*_), which are known parameters.

To overcome the strong coupling of **R** and **t** along the optical (*Z*_*c*_) axis in single‐plane calibration, we developed a multi‐plane ICP approach. The SMR tip traces a predefined Lissajous trajectory (*x*_*e*_ = *a* · sin *t*, *y*_*e*_ = *b* · sin *t*cos *t* for *t* ∈ [0, 2π]), where *a* and *b* control pattern width and length respectively, enabling adaptation to different FOV. This trajectory is executed at three distinct focal depths (*Z*_*c*1_, *Z*_*c*2_, *Z*_*c*3_) achieved by coordinated SMR bending, rotation, and 3‐DOF linear translation stage along *Z*_*c*_. The SMR end‐effector tip is visually tracked using OpenCV (Figure S5, Supporting Information). At each plane *k*, we collected *n* end‐effector positions {**E**_*k*, *i*_} = [*X*_*e*, *i*_, *Y*_*e*, *i*_, *Z*_*e*, *i*_]^*T*^ and corresponding image points {**I**_*k*, *i*_} = [*u*_*k*, *i*_, *v*_*k*, *i*_]^*T*^ (*k* = 1, 2, 3; *i* = 1, 2, ¨, *n*).

Our calibration pipeline employs three hierarchically refined stages: per‐plane coarse alignment, global linear initialization, and global ICP refinement. First, in the per‐plane coarse alignment stage, we independently apply ICP to each depth set to generate uniformly distributed points $\left\{E_{k,i}^{\prime }\right\}$. This critical preprocessing resolves trajectory mismatches caused by non‐uniform sampling while enabling parallel computation across the three planes, significantly improving computational efficiency and initial alignment quality. Second, the aligned points $\left\{E_{k,i}^{\prime }\right\}$ from all planes are merged for global linear initialization. By leveraging geometric constraints from multiple non‐coplanar planes, this stage decouples the inherent depth ambiguity in extrinsic calibration by least‐squares estimation of **R**_lin_ and **t**_lin_. This provides an optimal initial guess for the subsequent nonlinear optimization, avoiding error propagation from individual planes. Finally, global ICP refinement optimizes the transformation using the original kinematic points {**E**_*k*, *i*_} to preserve modeling accuracy, minimizing the spatial distance to image‐derived points {**P**_*k*, *i*_} (obtained by back‐projecting {**I**_*k*, *i*_} to normalized coordinates):

(5) $$\left[R,t\right]=\underset{R,t}{\arg\min}\sum_{k=1}^{3}\sum_{i=1}^{n}{∥R\cdot E_{k,i}+t-P_{k,i}∥}^{2}$$

initialized with [**R**_lin_, **t**_lin_] and constrained by **R** ^⊤^ **R** = **I**, det(R)=1. This hierarchical approach combines computational efficiency (through parallel plane processing), ambiguity resolution (via multi‐plane constraints), and accuracy preservation (by staged refinement) while effectively avoiding local minima common in direct nonlinear optimization.

**Figure 3:**
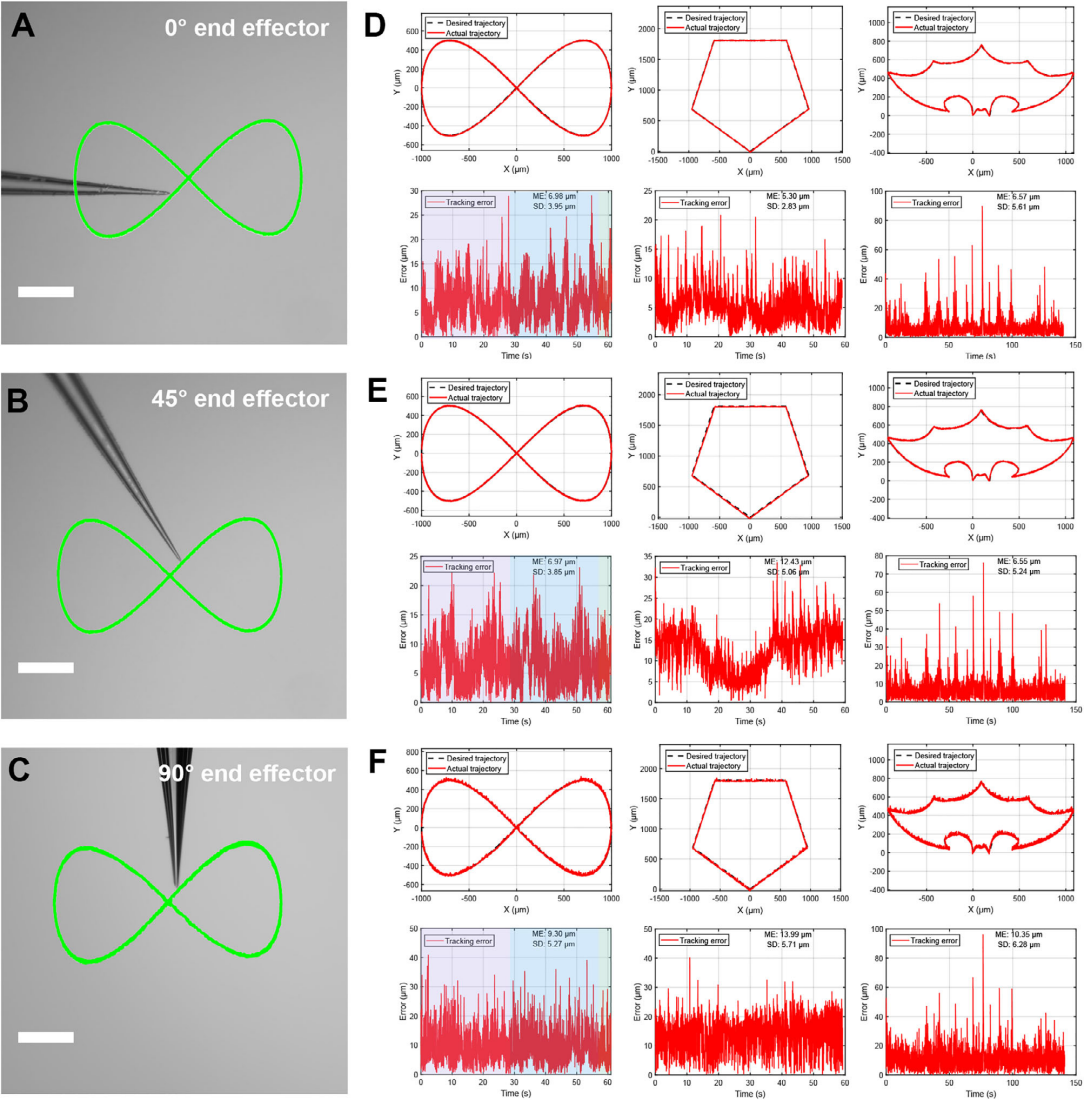
Multi‐angle validation of the fast calibration algorithm under varying configurations. A) Figure‐eight trajectory before calibration in the initial state. B) Bend 45° and re‐execute the calibration. C) Replace the end‐effector but bend it to 90° and re‐execute the quick calibration. (D–F) Positioning accuracy of three complex trajectories (figure‐eight, pentagon, bat‐shape) after calibration: D) initial state, E) 45° bending, F) end‐effector replacement with 90° bending. The results demonstrate robustness across geometric changes and large deflections. Scale bars: 200 µm.

To validate the efficiency and multi‐angle accuracy of fast calibration algorithm, we conducted two experiments. All tracking data were captured at 60 frames per second (fps) using a high‐speed camera (BioHD‐C20), while the SMR was controlled at 120 Hz (Figure S6, Supporting Information). Each trajectory consisted of ⩾ 1000 sampling points, ensuring high temporal resolution and robust trajectory reconstruction. First, calibration speed was assessed through 30 repeated trials. Our algorithm achieved 10 ± 1.9 s per calibration—a 90× improvement over traditional point‐to‐point methods (15 ± 2.6 min) while maintaining equivalent positioning accuracy. This efficiency enables practical deployment in time‐sensitive biological operations. Second, operational accuracy was evaluated across three test trajectories at different SMR bending angles (0°, 45°, 90°): continuous motion precision via figure‐eight paths (3 cycles, 4800 points), point‐to‐point targeting through pentagonal waypoints (1 cycle, 3500 points), complex trajectory tracking using bat‐shaped paths (1 cycle, 8500 points). At 0° (straight configuration), all trajectories achieved sub‐7 µ*m* accuracy (**Figure** **3**A,D). At 45° bending, continuous trajectories maintained similar high precision while pentagonal accuracy decreased to 12.43 ± 5.06 µ*m* due to tracking errors during directional transitions (Figure 3B,E). To test generalization capability, we performed 90° bending trials with different micropipettes, where all trajectories maintained precision sufficient for single‐cell manipulation (<14 µ*m*) (Figure 3C,F). Critically, the mean error across all configurations and trajectories remained below 14 µ*m*, meeting operational requirements for delicate single‐cell manipulations. The consistent error performance across all angular configurations demonstrates robust angular tolerance across the full workspace, validating the reliability of the calibration for complex 3D biological operations.

### 2.4 Validation of Operability and Adaptability

Biological micromanipulation is hindered by restricted operational angles, geometric constraints of culture vessels, and cross‐scale heterogeneity of biological targets. To address these challenges, we conducted two integrated experiments: functional operability testing to quantify angular accessibility and mechanical interaction characteristics during tip contact, and vessel adaptability validation across standardized containers.

**Figure 4:**
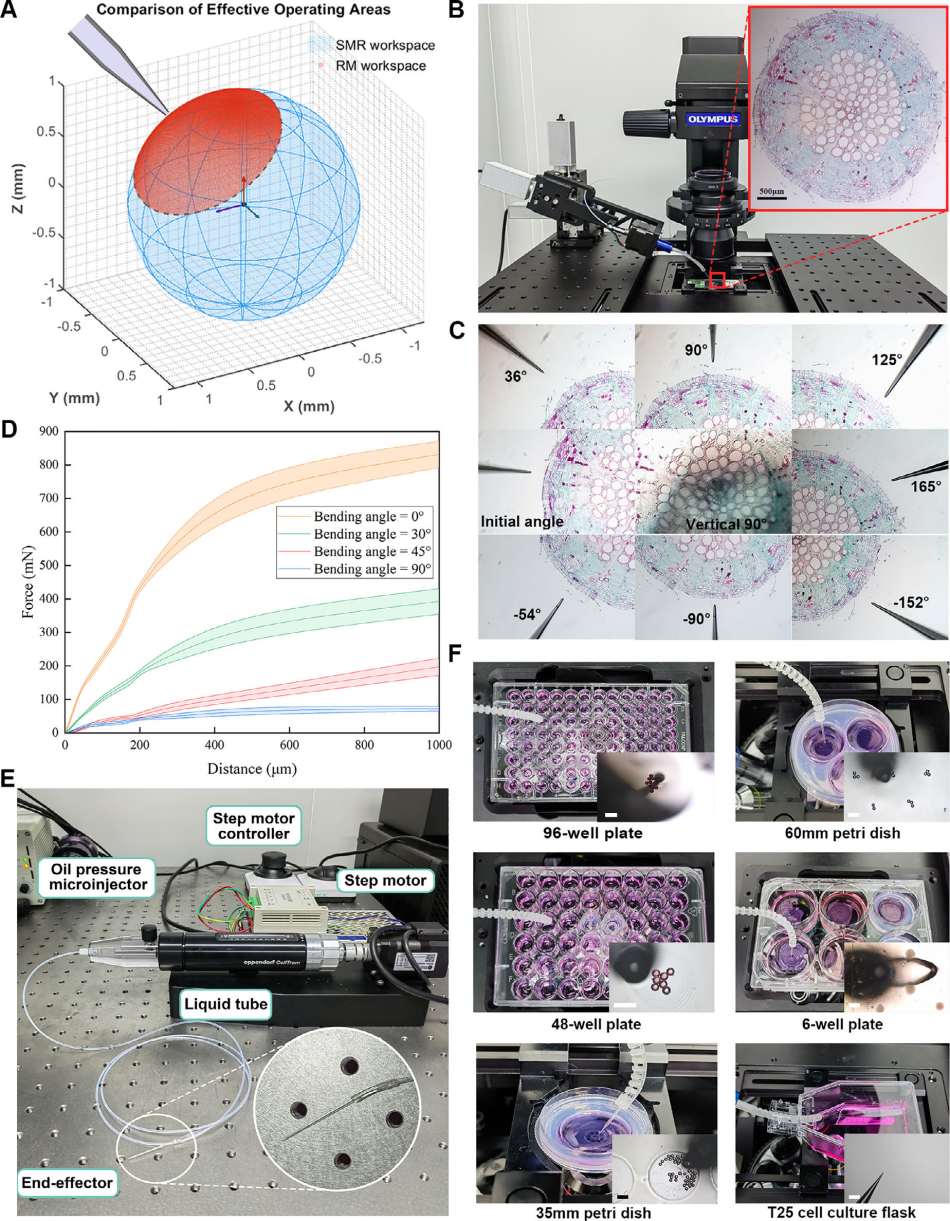
Adaptive performance characterization of SMR. A) Simulated workspace demonstrating up to a 6.8‐fold increase in accessible area for the SMR compared to conventional rigid manipulators (effective operating angle: 45°). B) Cotton root sample imaged under microscopy. Scale bar: 500 µm C) Full‐angle operability demonstrated through dynamic tip reorientation to accommodate spatial constraints. D) End‐effector force output as a function of displacement at four bending angles (0°, 30°, 45°, 90°). E) Components of the integrated microfluidic system: oil‐pressure microinjector (CellTram 4r), stepper motor, FEP tube, medical‐grade connector, and interchangeable end‐effectors. F) Demonstrated adaptability to six standard culture vessels: 96‐well, 48‐well, and 6‐well plates, 35‐mm and 60‐mm dishes, and T25 flasks. The SMR successfully manipulated 100 µm microbeads and cell spheroids in each vessel. Scale bars: 300 µm.

Conventional rigid manipulators (RMs) are fundamentally limited by their translational‐only mobility in standard linear stages. To quantitatively evaluate these limitations in biologically relevant scenarios, we model the accessible workspace around a target as a sphere of radius *S*, and define three geometric constraint categories based on sample type: free‐floating samples (full sphere, 4π*S* ^2^), adherent samples ($\approx \frac{2}{3}$ sphere), and samples in highly confined workspaces ($\frac{1}{2}$ sphere). The effective operational area *A*_*total*_ for rigid manipulators, including those with limited rotational adjustment (e.g., Narishige MKT‐1 with coaxial rotation angle), can be described as: *A*_*total*_ = 2π*S* ^2^[1 − sin (β − δ)]. The β is the effective operating angle and δ denotes any additional rotational capability. We compared our SMR against state‐of‐the‐art commercial manipulators (from Sutter, Eppendorf, Siskiyou, RWD, and Narishige) whose specifications are summarized in Table S2 (Supporting Information). Assuming an effective operational angle β = 45°, the SMR provides a 3.4 ×– 6.8 × improvement in accessible workspace area compared to these advanced rigid manipulators across the three constraint types, with the exact factor depending on environmental confinement and the adjustable angle of the manipulators (**Figure** **4**A; Note S2, Supporting Information). Experimental validation using a cotton root model (ϕ ≈ 3 mm) confirmed the practical advantage of this expanded workspace: the SMR robustly accessed all 10 predefined positions (Figure 4B,C). Two challenging cases highlighted its adaptive capability: a 180° bend combined with 60° rotation achieved a 165° viewing angle for shadow‐free visualization at a distal lateral site. Furthermore, a 60° bend compensated for the 30° inherent angle of the linear stage, enabling 90° vertical tip insertion and significantly reducing shear‐induced tissue damage. This reconfigurability allows for real‐time pose adjustment while maintaining full visual feedback.

Predictable force‐displacement behavior is essential for reliable micromanipulation (particularly injection) across varying bending angles. Force‐displacement curves were measured at angles of 0°, 30°, 45°, and 90° using a high‐precision analytical balance (BSA1245‐CW, Sartorius; 0.1 mg resolution). All force‐displacement measurements were performed using the same monolithically fabricated SMR. The 0–1000 µm displacement range was divided into 15 intervals, with 20 repeated measurements per interval to ensure data reliability. Before each of the 20 repeated measurements, the SMR was recalibrated to minimize systematic errors (Figure 4D). The test was conducted at a constant feed rate of 150 µm s^−1^, with intervals of less than 5 s to maximize simulation of automated injection scenarios. The antagonistic dual‐tube mechanism governs force modulation: at 0° bending, both the inner tube and the outer tube act simultaneously as rigid support components, delivering a maximum output of 830 ± 50 mN. As the bending increases to 30° and 45°, the outer tube transitions to an antagonistic role resisting deformation, progressively reducing force output to 392 ± 30 and 200 ± 20 mN, respectively. This trend continues at 90° bending, at which the force peaks at 70 ± 15 mN within a displacement of 500 µm, as the antagonist tube dominates structural stability. Importantly, SMR delivers 2.73 ± 0.69 mN at 10 µm displacement under 90° bending, a force sufficient for zebrafish oocyte puncture, which typically requires 0.7–1.2 mN.^[^ ^57^, ^58^ ^]^ Such predictable angle‐dependent mechanics enable precise microinjection calibration without compromising mechanical reliability.

To validate the adaptation of the SMR to typical vessels, the vessel selection criteria spanned a continuous size gradient (6.4–60 mm diameter), which collectively represented all key experimental parameters: volume (microliter to milliliter range), architecture (open vs. semi‐closed designs), and spatial complexity (2D to 3D culture microstructures). The 96‐well plates and T25 flasks represent opposing extremes in high‐throughput screening versus long‐term culture requirements, with intermediate vessels validating universal applicability. In addition, the SMR integrated a microfluidic system via the functional channel to enable rapid change of end‐effector (blunt tip for transfer and capture, sharp bevel for injection) to cope with different micromanipulations (Figure 4E and Experimental Section for design details). In 96‐well plates (ϕ 6.4 mm), 90° vertical bending enabled precise 100 µm fluorescent bead positioning (±14 µm), resolving high‐throughput manipulation constraints. For T25 flasks with 15° neck gradients, coordinated 60° bending ± 120° rotation achieved directional intervention in long‐term cultures through collision‐free wall access (Movie S2, Supporting Information). Intermediate‐scale operations demonstrated cross‐functional versatility: 60 mm dishes accommodated long‐range translation for 400–800 µm microstructure creation, while 85° bending in 35 mm dishes facilitated sequential bead placement into ring‐shaped formations (Movie S3, Supporting Information). Clusters transfer (280 ± 30 µm) between 400 µm and 800 µm microwells in 6‐well plates demonstrated reliable and non‐damaging handling of biological targets, confirming suitability for complex bioassembly tasks (Figure 4F).

### 2.5 Vitro Bioconstruction of Ring‐Shaped Structures

Circular biological structures, such as blood vessels, intestines, and renal tubules, are found widely in living tissues and offer unique advantages for modeling physiological microenvironments, constructing pathophysiological models, and investigating cellular mechanics and drug delivery efficiency.^[^ ^59^, ^60^, ^61^ ^]^ These biostructures are typically fabricated in vitro requiring external mechanical stimuli to guide cell self‐assembly into a 3D shape, with current approaches primarily utilizing biocompatible materials to provide structural scaffolds, such as PDMS,^[^ ^62^ ^]^ alginate,^[^ ^63^ ^]^ or agarose.^[^ ^64^, ^65^ ^]^ To obtain 3D cell spheres, we combined biochip technology to produce a biochip with an array of 127 uniform microwells based on 3% (w/v) agarose (for more detailed see Experimental section; Note S3, Supporting Information). The natural non‐adhesive properties of agarose were utilized to prevent cell‐substrate binding, enabling reliable production of free‐floating 293T cell spheres (230–250 µm in diameter) within 24 h for standardized comparative studies (Figure S7, Supporting Information). In 3D cell culture, the construction of physiologically relevant ring‐shaped structures poses a greater challenge. These complex structures require the precise multi‐angle spatial arrangement of individual cell clusters to reproduce tissue‐level organization and lumen formation. This capability for omnidirectional manipulation under spatial constraints remains fundamentally lacking in current robotic platforms.^[^ ^43^, ^66^, ^67^ ^]^

**Figure 5:**
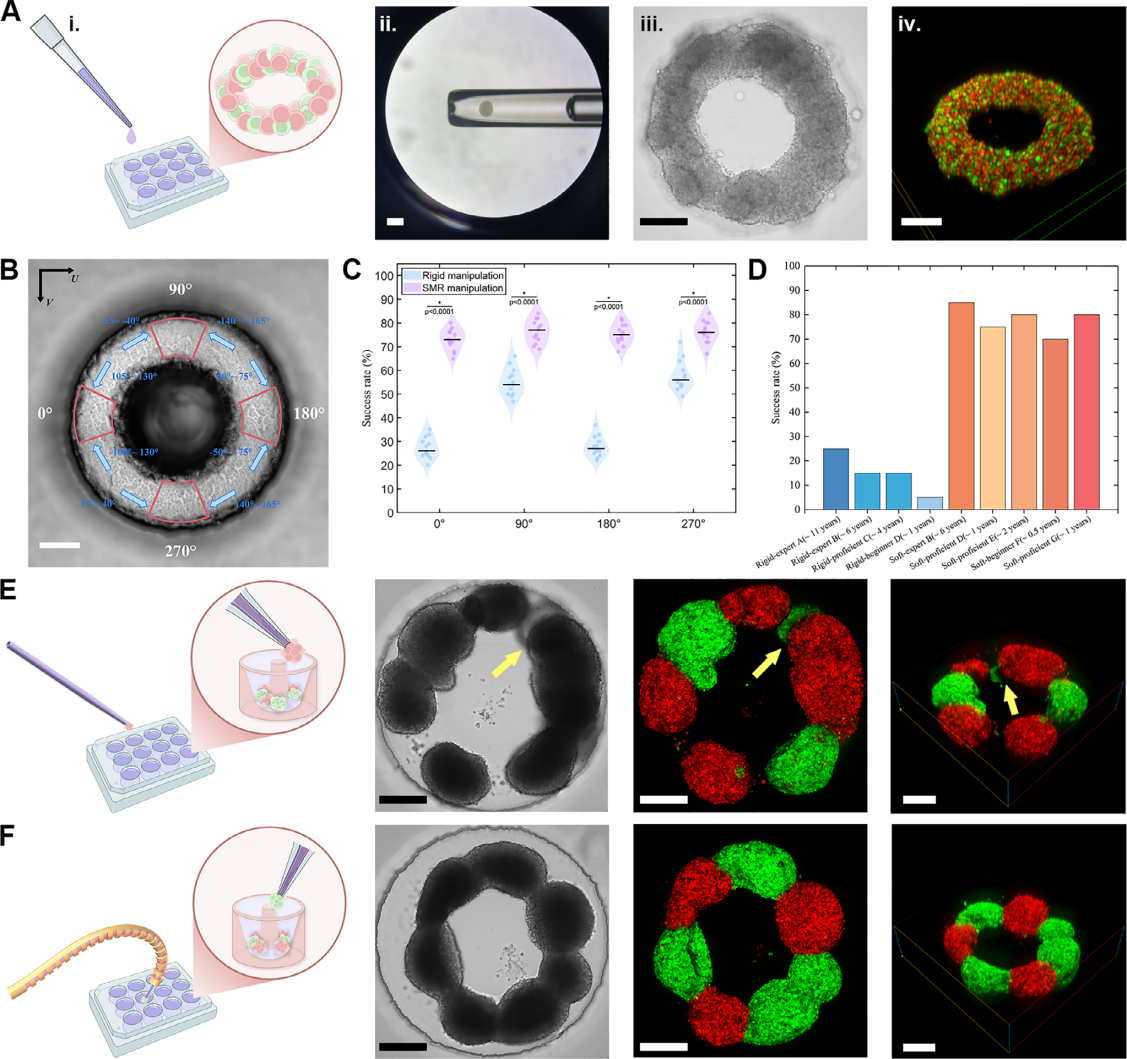
Performance assessment of cell cluster patterning by soft micromanipulation compared to traditional methods (rigid micromanipulation and manual pipette manipulation). A) Manual pipette manipulation situations and results, including i) schematic of the manual random droplet deposition method, ii) aspiration of cell clusters by a modified pipette, iii) manual manipulation of ring formation in bright field and iv) 3D fluorescent voxel image of the manually formed ring. B) The ring‐shaped chip is functionally divided into eight distinct regions. Cell spheres located in four key regions were positioned using specific approach angles, which are defined relative to the U‐axis of the image coordinate frame (*O*_*i*_–*UV*). C) Comparative success rates of soft vs rigid micromanipulation at critical ring positions (0°, 90°, 180°, 270°). Violin plots (N=100 independent placements per position; *p < 0.0001, two‐tailed *t*‐test) demonstrate significant performance differences across all angles. Thirteen operators performed placements under identical conditions. D) Comparison between ring formation experiments by seven operators with different micromanipulation experience (operators A, B, C, D, E, F, G) using both a soft micromanipulation robot and a rigid robotic arm. (Number of times the cell ring was placed: N=20) E) Failed ring formation attempt using rigid micromanipulators, showing cell cluster stacking (yellow arrows) and displacement due to angular inflexibility and excessive contact forces. F) Precise arrangement of seven 293T cell clusters (3 mCherry‐labeled and 4 GFP‐labeled) into a predefined ring pattern using the soft micromanipulation robot, achieving positional accuracy within 20 µm. The robot's adaptive bending capability enabled direct placement at the groove bottom without structural damage. All scale bars: 300 µm.

The manual pipette manipulation approach, using modified 10 µL pipettes with 300 µm tips, demonstrated critical limitations in precision positioning (**Figure** **5**A; Figure S8, Supporting Information). Although these tools could successfully aspirate individual 293T cell spheroids, physiological hand tremors (50–200 µm amplitude) led to positioning errors greater than 200 µm during deposition.^[^ ^68^, ^69^, ^70^ ^]^ This precision constraint forced operators to resort to stochastic droplet deposition methods, where cell suspensions were randomly dispersed in hanging droplets. Although random deposition occasionally achieved ring‐shaped cell assemblies, these structures exhibited disorganized cell‐cell contacts and proved largely incapable of forming reproducible ring‐shaped biostructures suitable for physiologically relevant studies (Figure 5 Aiii–iv).

To evaluate the SMR's ability to construct complex biological architectures, we compared its multi‐angle manipulation performance against a rigid micromanipulator restricted to fixed‐angle access in two experiments. In the first experiment, single‐cell spheroids were precisely positioned at four predetermined locations (0°, 90°, 180°, 270°) within a ring‐shaped biochip (Figure 5B; Figure S1B, Supporting Information). These positions were chosen to present significant spatial challenges due to obstructing chip walls, thereby testing the manipulator's ability to access targets from non‐axial angles. The second experiment focused on the construction of complete, continuous ring‐shaped assemblies using dual‐fluorescent (mCherry/GFP) 293T cell spheroids. We quantified performance in three critical parameters: spatial positioning accuracy, incidence of biochip damage, and structural integrity of the final bioassembly. Throughout both experiments, we recorded the incidence of damage to fragile cell spheroids and the integrity of the biochip substrate to assess environmental adaptability.

Thirteen operators participated in the cell placement experiment, including two experts (6–11 years), two proficient operators (2–4 years), three novices (0.5–1 year), and six individuals with no prior experience. The seven operators with prior experience (i.e., experts, proficient, and novices; 0.5–11 years of experience) performed the cell ring‐shaped assembly experiments. For the placement experiment, each operator performed 100 replicate positioning attempts at the four critical positions. While the rigid manipulator maintained stable positioning accuracy, its overall success rate remained suboptimal (Figure 5C). The fixed‐angle operational mode caused biochip damage in ≈24% of attempts, with particularly high incidence at 0° and 180° positions. Such damage not only compromised the cultured microenvironment, but also introduced uncontrolled variables that affected the stability of biological experiments. For collision‐free operations, the rigid manipulator depended on a hover‐and‐release method. The effectiveness of this method proved to be highly sensitive to uncontrollable parameters including medium viscosity, individual sphere mass and release height, which are difficult to compensate for these instabilities by precise control. In contrast, the SMR significantly enhanced placement success through multi‐angle adjustment, achieving success rates of 73–77% across all angles, compared to 26–56% for the rigid manipulator. The improvement was statistically significant at all positions (t‐test: p = 3.25 × 10^−19^ at 0°, p = 2.52 × 10^−10^ at 90°, p = 4.97 × 10^−20^ at 180°, and p = 8.42 × 10^−9^ at 270°). For the challenging 0° position, an approach angle of 105°–130° relative to the *U*‐axis in the image (achieved via 75°–100° of bending combined with 10°–45° of rotation) was required, which increased the success rate by 45%. Similarly, for the 180° position, an operating alignment of 15°–40° provided a clear FOV to prevent collisions. For the 90° and 270° positions, the SMR's tip was deliberately aligned at an inclined angle of 15°–40° relative to the groove. This approach reduced hydrodynamic disturbances and improved the placement success rate to 80%, significantly exceeding the performance of rigid micromanipulators.

Ring assembly experiments employed agarose ring‐shaped biochips to ensure microenvironmental consistency. GFP‐ and mCherry‐labeled cells were kept in separate areas to prevent fluorescence interference during manipulation or culture while maintaining sufficient proximity for efficient cluster transfer (Note S4, Supporting Information). The Rigid manipulator operators (A‐D, 20 trials each) achieved success rates below 30%, showing a strong experience dependency (Figure 5D). Fixed‐angle constraints necessitated stochastic deposition methods, resulting in 25% cluster overlap and 32% biochip damage that deformed ring geometry. Critically, these conditions led to cell cluster placement errors of more than ± 20 µm, which would increase the intercluster distance beyond the effective adhesion range leading to ring formation failure (Figure 5E; Note S4, Supporting Information). In contrast, operators (B, D–G) using the SMR demonstrated noticeably improved performance across multiple metrics (Figure 5D). Success rates reached 85% for the expert operator, 80% for the proficient operator, and 70–85% for novice operators, indicating consistent performance regardless of experience level. Biochip damage was reduced to just 3%, and cluster overlap was effectively eliminated owing to the robotic omnidirectional access (Figure 5F). Furthermore, ring circularity was significantly enhanced through minimized manipulation steps, contributing to more precise and reliable structure assembly (Movie S4, Supporting Information). Collectively, these results demonstrate that the SMR's real‐time adaptive capability, which is enabled by multi‐angle compensation and initial pose adjustment, effectively overcomes the inherent limitations of conventional rigid manipulators in complex 3D bioconstruction. This capability ensures precise, reliable operations while preserving the integrity of the microenvironment.

### 2.6 Biomedical Applications of Omnidirectional Soft Micromanipulation Robot

**Figure 6:**
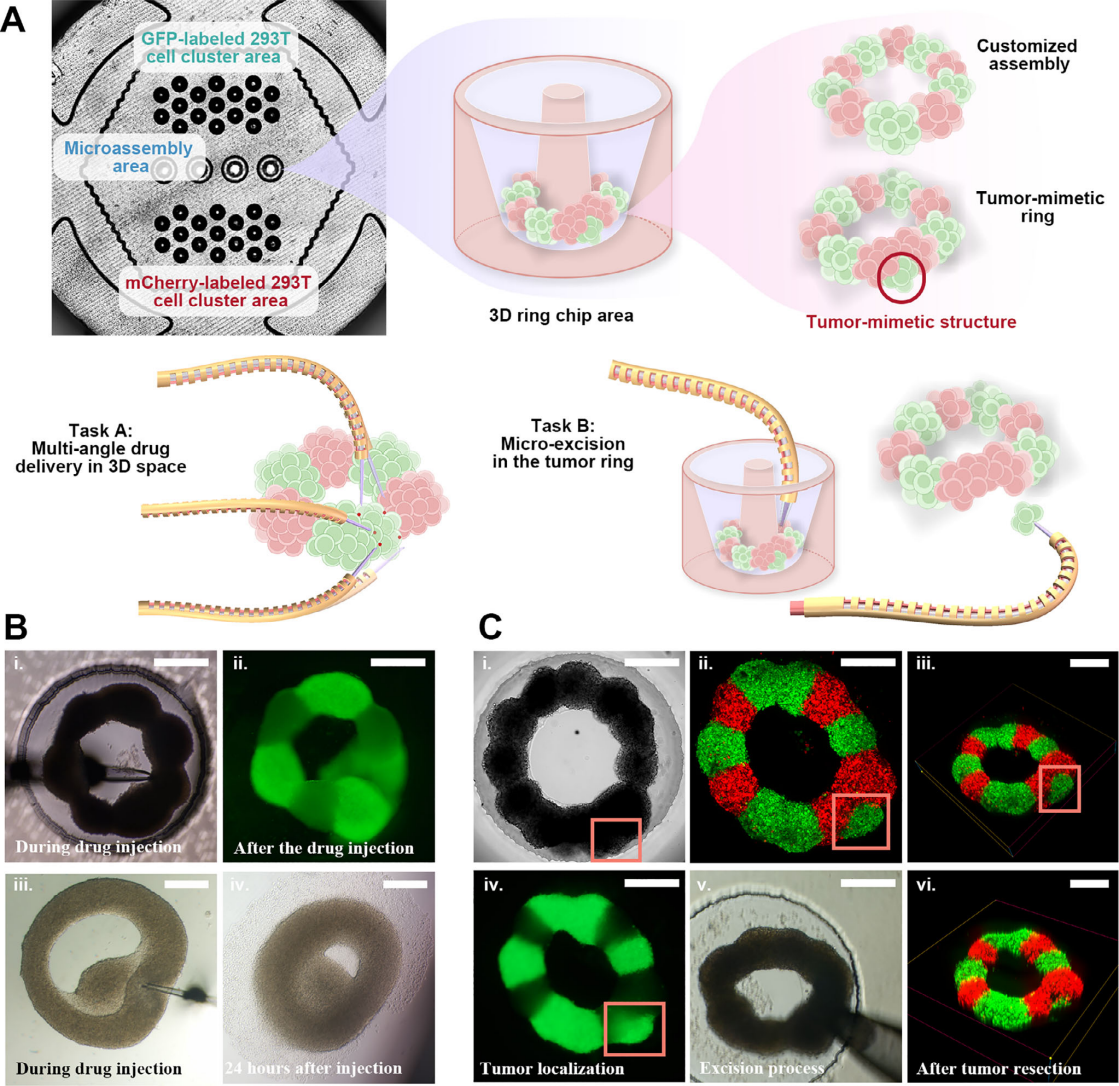
Micromanipulation Surgery. A) The SMR can selectively perform different micromanipulation tasks. B) Multi‐angle drug injection microsurgery. i) Bright field image of the drug microinjection process. ii) 293T cell spheres are injected with 75% alcohol at the moment of disintegration. iii) 293T cell rings were injected with different concentrations of 5‐FU. iv) The site of 5‐FU drug injection exhibited growth inhibition. C) SMR resection of the tumor in the ring‐shaped assembly. i) Bright‐field image of a ring‐shaped tumor assembly. ii) Bionic tumor model microscopic fluorescent image showing GFP‐labeled tumor spheroids (100 µm in diameter) embedded in concentric healthy cell layers expressing mCherry, forming a mimic tumor structure (280 × 150 µm). iii) 3D voxel image of the bionic tumor model. iv) Tumor localization under fluorescence microscopy. v) The process of resecting the tumor with micropipette. vi) 3D voxel fluorescence image of a bionic tumor model after resection with a remnant <10 µm. All scale bars: 300 µm.

In this section, we conducted a series of experiments with the developed SMR for demonstrating omnidirectional manipulability, task adaptability, and multifunctionality, which cannot be realized by conventional micromanipulators. These experiments systematically demonstrate the ability of robots to perform advanced tasks, including capture, transfer, injection, and resection in fragile, heterogeneous microenvironments while maintaining the structural integrity of biological samples and their surrounding matrices (**Figure** **6**A).

Achieving high targeting accuracy in localized drug delivery is essential for reliable therapeutic evaluation. However, mainstream drug screening approaches typically rely on fixed‐angle, high‐throughput devices that deliver drugs via bulk administration into multi‐well plates, resulting in uniform bath application across entire tissue or cell populations. In contrast, the presented SMR enables a new method for precision pharmacological testing, allowing spatially controlled, cell‐level drug delivery. This capability addresses a critical limitation of conventional batch administration methods, which fail to provide spatially resolved pharmacodynamic information–particularly in anti‐cancer drug screening, where distinguishing drug effects between pathological and healthy tissues is paramount. We developed a multi‐angle drug delivery robot leveraging the flexible operation framework established in Section 2.5. We constructed alternating GFP‐ and mCherry‐labeled cell rings (150 µm radius) assembled from 200 µm 293T fluorescent cell clusters (Figure 6B‐i), enabling 3D spatially programmed drug delivery across multiple planes (80 µm Z‐axis spacing). The SMR employed filamented borosilicate glass microelectrodes (1.0 mm × 0.5 mm) pulled into ≈5 µm inner diameter tips with a long taper, ensuring smooth fluid control and precise tissue penetration while minimizing collateral damage. The SMR demonstrated exceptional adaptability in localized drug delivery, enabling spatially and temporally controlled pharmacological interventions with high resolution. Using dual‐modality (bright‐field and fluorescence) imaging, we monitored real‐time drug distribution and tissue response during delivery. Three pharmacologically distinct drugs were tested to represent diverse therapeutic scenarios: 75% ethanol as a rapid‐lytic drug, Zeocin (10 mg mL^−1^) as a slow‐diffusing antibiotic, and 5‐fluorouracil (5‐FU) as a dose‐dependent chemotherapeutic. Ethanol was delivered via high‐pressure pulses (2 µL s^−1^), resulting in immediate structural disintegration of target cells (Figure 6Bi,ii). In contrast, Zeocin was administered in a low‐pressure, intermittent mode (0.1 µL s^−1^ with 2 s intervals) to facilitate gradual diffusion while minimizing mechanical disruption. For 5‐FU, a concentration gradient (100–1000 µM) revealed a threshold effect: concentrations below 600 µM induced no significant growth inhibition, whereas 800 and 1000 µM treatments led to marked suppression of 293T cell expansion at the injection sites (Figure 6Biii,iv). Throughout these procedures, the SMR's dynamic reconfiguration, which was enabled by adjustable bending (15°–120°) and rotation (± 90°), ensured precise navigation through complex, layered architectures, with each injection completed within 30 s. A cell spheroid was additionally assembled near the central injection site within the microring to assess drug diffusion and penetration from the delivery point. These results demonstrate the SMR's capability to deliver diverse pharmacological agents with programmable spatio‐temporal precision, supporting its potential for quantitative drug efficacy studies.

Cancer research and drug development critically depend on precise tumor excision techniques to investigate tumor‐stroma interactions, evaluate treatment responses, and explore metastatic mechanisms.^[^ ^71^, ^72^ ^]^ Traditional methods like mechanical scraping or enzymatic digestion invariably damage surrounding healthy tissues. Here, the SMR enables precision microsurgery within confined 3D microenvironments using biomimetic tumor models (Figure 6 Ci–iii). We programmably embedded GFP‐labeled “tumor” clusters (100 µm diameter, half the size of normal cell masses) within mCherry‐expressing healthy cell rings. After 24h culture under biochip confinement and gravitational settling, these developed into mature tumor‐mimetic structures (280 × 150 µm) with authentic cell adhesions. Tumors were accurately localized via combined bright‐field and fluorescence imaging (Figure 6C‐iv). Leveraging the SMR's cellular precision, excision used borosilicate micropipettes (10 µm inner diameter, 3–5 mm medium‐taper) via multi‐point progressive suction, optimizing separation efficiency and clog prevention (Figure 6C‐v). This strategy effectively prevented mechanical damage to healthy tissues while allowing real‐time monitoring and dynamic parameter adjustment during microsurgery. Post‐operatively, 3D reconstruction and volumetric analysis of 3D fluorescence image stacks confirmed the high precision of the resection (Z‐step = 4.02 µm). Approximately 98.4% of the tumor‐mimetic mass was successfully removed, while over 99% of the enclosed healthy tissue (red fluorescent 293T cells) was preserved intact. This demonstrates the capability of our approach to achieve thorough tumor excision with exceptional sparing of adjacent healthy structures (Figure 6C‐vi).

## 3 Conclusion

In this work, we presented soft micromanipulation robot (SMR) to address three major challenges in biological micromanipulation—cross‐scale target manipulation, constrained workspace navigation, and integrated multimodal operations. Our hollow nested agonist‐antagonist mechanism suppresses bending instability, while the photopolymerized integrated structure eliminates torsion induced by force imbalance. This design enables “full angle” manipulation (± 180° bending, 360° rotation) within microscope FOV when integrated with a 3‐DOF linear translation stage. Through rigorous trajectory tracking tests (pentagon, figure‐eight, and irregular bat paths), SMR demonstrated ± 14 µm positioning accuracy across critical orientations (0°, 45°, 90°). The SMR achieved a 6.8‐fold increase in effective workspace compared to rigid manipulators, as demonstrated in tasks including non‐occluding multi‐angle access to the cotton root and microbead transfer across diverse labware (Petri dishes, multiwell plates, culture flasks, and biochips). This enhanced operational range minimizes collision risks while maximizing usable space in confined workspaces. We successfully executed integrated operations including aspiration, transfer, programmable assembly, microinjection, and cutting across targets ranging from fluorescent microbeads to 293T cell clusters. Crucially, we fabricated ring‐shaped biostructures from GFP‐ and mCherry‐labeled 293T cells within confined biochip, creating spatially organized cellular systems for drug screening. To demonstrate its microsurgical capabilities, SMR executed two high‐precision surgery experiments. The first involved multi‐angle microinjection with 5 ± 2.4 µm accuracy, utilizing adaptive delivery modes: low‐pressure continuous release for targeted single‐point treatment with Zeocin solution, and high‐pressure pulsatile injection for multi‐point ablation of specific cell spheres using 75% ethanol. The second application demonstrated high‐precision tumor‐mimetic ring excision, where volumetric analysis confirmed the removal of 98.4% of the target mass while sparing adjacent healthy cells. This precision, combined with its adaptive injection system for localized drug delivery, facilitates personalized therapy studies.

Although the current resin‐based prototype exhibits delayed elastic recovery during intensive cyclic operations, this is an acceptable cost‐benefit compromise for core functionality. The progressive reduction in realized displacement over repeated actuation cycles is the primary consequence of cumulative viscoelastic relaxation and residual strain, leading to motion inaccuracies and force variability. To mitigate this effect in real time, we employ a vision‐based feedback control strategy to actively compensate for displacement deviations (Figure S9, Supporting Information). By leveraging the high‐resolution imaging, the system detects and corrects positional errors online, ensuring consistent operational accuracy across cycles. Alternatively, performance can be sustained through on‐demand DLP printing for task‐specific fabrication, or by substituting the structure with superelastic materials (e.g., Nitinol) to enhance reusability and reduce recovery delays. This high‐precision multi‐angle operation capability holds promise for applications in small model organisms, including organoids, zebrafish larvae, and Drosophila larvae, enabling targeted interventions on delicate anatomical sites such as gonads, heart, and sensory organs. With the continued development of multi‐agent intelligence and advanced sensing technologies, this omnidirectional micromanipulation robotic system capable of integrating and processing multi‐modal data will become a robust and scalable platform for intelligent micromanipulation research in the future.

The assembled ring‐shaped structures exhibited robust architectural stability within the biochips, maintaining their defined geometry for over seven days without detectable deformation. Notably, a necrotic core consistently emerged at the center of each structure by day 3 to 5, likely due to diffusion limitations of oxygen and nutrients arising from the high cellular density.^[^ ^73^, ^74^, ^75^ ^]^ Upon release into standard culture, structural integrity gradually degraded: cells migrated outward within 24 h, forming a peripheral halo, and the ring flattened by day 3, highlighting the necessity of physical confinement for 3D organization. To probe pharmacological responses, we performed targeted 5‐FU injections (800 µM, 0.5 µL) into 200 cell rings, with control groups receiving 0.5 µL medium (10%FPS + DMEM). Although high‐concentration 5‐FU effectively inhibited the local growth of the ring structure as expected, the ATP assay results were counterintuitive, with RLU values slightly higher in the drug‐injected group than in the control group. We hypothesize that this effect may result from either competitive release, in which the elimination of drug‐sensitive, weaker cells benefits healthier neighbors, or mild hormesis, whereby low‐level 5‐FU transiently enhances metabolic activity. This unexpected response reveals a complex spatial dynamics of drug action that cannot be studied with conventional techniques.

## 4 Experimental Section

### 3D‐SLA Printing

The SMRs were fabricated using a Form 3B+ stereolithography (SLA) printer (Formlabs, Somerville, USA). The initial prototypes used for functional characterization (e.g., kinematic performance, workspace analysis) were printed using Clear Resin (Formlabs). For all biological experiments, to ensure full biocompatibility, the SMRs were fabricated using BioMed Clear (Formlabs), a medical device material certified to be non‐cytotoxic according to ISO 10993‐5. To achieve high precision and surface quality, the layer thickness was set to 25 and 50 µm, respectively. Slicing files were generated using PreForm software (Formlabs, Somerville). A rigorous post‐processing protocol was applied to eliminate uncured resin and sterilize the devices for cell culture experiments. After printing, the structures were first washed in anhydrous ethanol for 20 min. They were then treated for 10 min in fresh anhydrous ethanol in an ultrasonic bath to thoroughly remove any residual monomers. Finally, for sterilization, the devices were immersed in a 75% ethanol solution for 30 min, rinsed 2‐3 times with sterile deionized water, and allowed to dry under a biological safety cabinet prior to use.

### Cell Culture

The 293T cell lines (GNHu17) were obtained from the Cell Bank of the Center for Excellence in Molecular Cell Science, Chinese Academy of Sciences. These cells were maintained in Dulbecco's Modified Eagle Medium (DMEM) supplemented with 10% fetal bovine serum (FBS) at 37°C in a humidified atmosphere containing 5% *CO*_2_.

### Agarose Gel Preparation

An appropriate amount of agarose (Baygene Biotechnologies,Shanghai, China) was weighed according to the ratio of 0.03 grams per milliliter of PBS solution and added to PBS (phosphate‐buffered saline) solution (Boster Biological Technology, China) with a pH of 7.4 provided, adjusting the final concentration of agarose in the solution to 3% (w/v). The mixture was poured into a clean microwave‐safe container and heated in a microwave oven until it reached boiling, with intermittent stirring to ensure even dissolution of the agarose. After boiling, the solution was further heated in the microwave for ≈2 min to ensure thorough dissolution.

### Modular Microfluidic Delivery System

The fluidic device consists of three key components: 1) a fluid delivery channel made of 0.19‐mm‐inner‐diameter fluorinated ethylene propylene (FEP) tube, 2) custom‐designed modular connectors fabricated from medical‐grade modified polyvinyl chloride (PVC) with 0.15 mm inner diameter, and 3) interchangeable micropipettes. The specially engineered connectors feature asymmetric expansion with one end expanded to 2 mm for secure FEP tube connection and the opposite end to 1 mm for versatile micropipette adaptation. These micropipettes can be fabricated using a micropipette puller (P‐97, Sutter) to produce customized tips for specific functions such as cell capture, microinjection, and sample transfer. For precise fluid control, the integrated system is coupled to an oil‐driven microinjector (CellTram 4r Oil, Eppendorf), which is actuated by a stepper motor to enable accurate manipulation at the microscale.

### Microarray Chip Fabrication

The microarray chips were fabricated by pouring molten 3% (w/v) agarose in PBS at ≈60°C into sterile Petri dish placed on a precisely leveled surface within a biosafety cabinet. While maintaining the agarose temperature above 40°C to prevent premature gelation, a light‐cured 3D‐printed mold containing 800 µm diameter microvia arrays and ring patterns was carefully pressed into the solution with uniform pressure to create features with 500 µm depth. The assembly was left undisturbed at room temperature for 30 minutes to allow complete solidification, after which the mold was gently removed to reveal the patterned agarose structure. The fabricated chips were immediately sealed in sterile PBS and stored at 4°C until use, with final sterilization achieved through 30 min of UV irradiation immediately prior to cell seeding, ensuring both structural fidelity and sterility for subsequent biological applications.

### Microscopy Imaging and Micromanipulation System

Fluorescence analysis was performed using a spinning‐disk confocal microscope system (IX83, Olympus, Japan) coupled with the cellSens Dimension image acquisition and analysis software. For dual‐color labeled 293T cells, mCherry fluorescence was excited by a 561 nm laser (emission band: 575–625 nm, exposure: 800 ms) while GFP was excited by a 488 nm laser (emission band: 510–550 nm, exposure: 300 ms). All imaging procedures maintained consistent laser power at 100 mW to ensure data comparability.

Micromanipulation experiments were conducted using an inverted microscope system (IX73P1F, Olympus, Japan) equipped with a high‐sensitivity fluorescence camera (BioHD‐C20), enabling real‐time fluorescent imaging guidance for precise manipulation procedures.

Large‐area imaging of biochips and microarray chips utilized a high‐precision motorized XY stage (H117E1XD, ProScan, UK; positioning accuracy: ±0.5 µm) for target localization, with multi‐field image registration achieved through the auto‐stitching function of the cellSens Dimension software.

3D voxel reconstruction was performed through Z‐axis serial sectioning, beginning with the precise definition of top and bottom focal plane coordinates at 0.01 µm Z‐step precision, followed by sequential acquisition of optical sections at 4.2 µm intervals (typically 60–75 layers depending on sample thickness), and culminating in the generation of high‐fidelity 3D voxel models using the advanced reconstruction algorithms embedded in the cellSens Dimension software platform (Olympus, Japan).

### Statistical Analysis

All quantitative data are presented as mean ± SD. In the biological manipulation experiments, performance comparisons between the rigid manipulator and the SMR were conducted using independent two‐sided two‐sample t‐tests. For the programmable cell loop formation task, a total of 7 operators with varying experience levels (2 experts, 2 proficient, and 3 novices) each performed 20 attempts, and their success rates were compared between the two devices. For the cell sphere placement task at four key angular positions (0°, 90°, 180°, and 270°), the performance datasets of the two devices were statistically compared, with each group containing data from 13 operators (n = 13), comprising 2 experts (>6 years of experience), 2 proficient (2–4 years), 3 novices (0.5‐1 years), and 6 inexperienced users. All statistical analyzes were performed using the ttest2 function in MATLAB R2021b software, with a significance level set at α = 0.01.

## Conflict of Interest

The authors declare no conflict of interest.

## Supporting information

Supporting Information

Supplemental Movie 1

Supplemental Movie 2

Supplemental Movie 3

Supplemental Movie 4

## Data Availability

The data that support the findings of this study are available from the corresponding author upon reasonable request.
