# Supplementary material for: Soft Micromanipulation Robot for Real‐Time Adaptive Multimodal Operation
Source: Adv Sci (Weinh). 2025 Oct 21;13(2):e15784. doi: 10.1002/advs.202515784 (PMC12786275; doi:10.1002/advs.202515784)
Supplement: Supplementary file 1 — Supporting Information [file ADVS-13-e15784-s003.pdf]

Real-Time Adaptive Omnidirectional Soft Micromanipulation Robot

Zhuowei Li, Xiaotian Lin, Zhoujie Zhu, Yibo Zhu, Yanping Zhou, Jing Li, Chris Gerada, He Zhang*, and Songlin Zhuang*

Yongjiang Laboratory, Ningbo, 315201, Zhejiang, China
University of Nottingham Ningbo China, Ningbo, 315100, China and
Power Electronics, Machines and Control Research Group,
University of Nottingham, Nottingham, NG8 1BB, United Kingdom

(Dated: September 24, 2025)

Supporting Information

Supporting Note 1: Kinematics of soft micromanipulation robots

Here, we describe in detail the displacement of the linear motor T with respect to the bending angle and the position in spatial coordinates. The distance traveled by the motor is defined as d equal to the distance traveled by the inner tube. L_i is the initial length of the inner tube, and its uncut portion also remains constant during movement. $L_{i'}$ denotes the actual length of the inner tube through the outer tube, which varies with movement. The relationship between them can be expressed as:

$$d = L_i - L_{i'} \tag{1}$$

The initial length L_i can be expressed by the spacing c between the notches of the inner tube and the length w of the notches. Where subscript i denotes the inner tube, o denotes the outer tube, and j denotes the jth section unit.

$$L_i = \sum_{j=1}^{n} (c_{i,j} + w_{i,j}) \tag{2}$$

The length $L_{i'}$ through the inner tube is the sum of the uncut portion of each unit $c_{o,j}$ and the length $L_{i',j}$ of the cut through the outer tube.

$$L_{i'} = \sum_{j=1}^{n} (c_{o,j} + L_{i',j}) \tag{3}$$

where $L_{i',j}$ can be found by geometric relations:

$$\begin{cases} L_{i',j} = r_{i,j} w_j \kappa_{o,j} \\ \Delta X_j = r_{o,j} - r_{i,j} \end{cases} \tag{4}$$

where r_o, r_i, κ_o and κ_i denote the radius of curvature and curvature of the inner and outer tubes, respectively. Where ΔX_j is the distance between the center of mass of the inner and outer tubes.

$$d = \sum_{j=1}^{n} \Delta X_j w_j \kappa_{o,j} \tag{5}$$

The centerline bending curvature for a soft micromanipulation robot can be expressed as:

$$\kappa_j = \frac{\kappa_{o,j}}{1 - \gamma_{o,j}\kappa_{o,j}} \tag{6}$$

As the bending changes, the length of the center curve can be expressed as:

$$l_j = \frac{w_j}{1 + \gamma_{o,j}\kappa_j} \tag{7}$$

The bending angle for the jth bending unit can be calculated as:

$$\theta_j = \kappa_j l_j \tag{8}$$

The transformation from the base of notch j to the base of notch $j+1$ consists of a variational curvature bending transformation

$$\mathbf{T}_{notch,j} = \begin{bmatrix} \cos(\kappa_j l_j) & 0 & \sin(\kappa_j l_j) & (1-\cos(\kappa_j l_j))\,\kappa_j^{-1} \\ 0 & 1 & 0 & 0 \\ -\sin(\kappa_j l_j) & 0 & \cos(\kappa_j l_j) & \sin(\kappa_j l_j)\,\kappa_j^{-1} \\ 0 & 0 & 0 & 1 \end{bmatrix} \tag{9}$$

The rigid segment matrix transformation of the jth unit of the uncut part is:

$$\mathbf{T}_{rigid,j} = \begin{bmatrix} 1 & 0 & 0 & 0 \\ 0 & 1 & 0 & 0 \\ 0 & 0 & 1 & c_j \\ 0 & 0 & 0 & 1 \end{bmatrix} \tag{10}$$

The matrix transformation of the length of the connecting layer at the end beyond the rigid segment is:

$$\mathbf{T}_{z,b} = \begin{bmatrix} 1 & 0 & 0 & 0 \\ 0 & 1 & 0 & 0 \\ 0 & 0 & 1 & z_b \\ 0 & 0 & 0 & 1 \end{bmatrix} \tag{11}$$

Z_e represents the lengths of the different end-effectors, and their matrix transformation is:

$$\mathbf{T}_{z,e} = \begin{bmatrix} 1 & 0 & 0 & 0 \\ 0 & 1 & 0 & 0 \\ 0 & 0 & 1 & z_e \\ 0 & 0 & 0 & 1 \end{bmatrix} \tag{12}$$

Integrating the above equations gives an expression for the radius of rotation:

$$\mathbf{u}_{R(d)} = (z_b + z_e) \prod_{j=1}^{n} \sin\theta_j + \prod_{j=1}^{n} \left(\frac{1 - \cos\theta_j}{\kappa_j} + c_j \sin\theta_j \right) \tag{13}$$

Supporting Note 2: Detailed analysis of accessible workspace improvement

1. Three categories of biological models

TABLE I. Comparison of operable space improvement for different biological models

**Biological Model Type**	**Description**	**Operable Area**	**Improvement Factor**
Free-floating models	Suspended cells, cell spheres, zygotes, free organoids	$4\pi S^2$	6.8×
Adherent models	Zebrafish, attached embryos, surface-adhered cell spheres	$\frac{2}{3} \cdot 4\pi S^2$	4.5×
Structurally constrained models	Samples in micro-wells, organ-on-chip devices, bioreactors	$2\pi S^2$	3.4×

To quantitatively evaluate the spatial accessibility of different manipulators, we model a biological sample as a sphere of radius R. The surface of this sphere represents all possible points where a manipulator's end-effector (e.g., a micropipette tip) might need to make perpendicular contact with the sample to perform an operation (e.g., injection, aspiration). The fundamental capability of a manipulator is thus defined by the fraction of this spherical surface it can access.

Physical constraints inherent to biological experiments often limit access to only a portion of this sphere. We categorized these constraints into three biologically relevant scenarios (Figure 1A-C):

1. Free-floating models (e.g., suspended cells, organoids, zygotes): Samples are suspended in a medium, allowing theoretical access to the entire spherical surface area ($4\pi R^2$).

2. Adherent models (e.g., zebrafish embryos, surface-attached cells): Samples are adhered to a substrate (e.g., a dish bottom), which physically obstructs access to approximately one-third of the sphere. The effectively accessible area is reduced to $\approx \frac{2}{3} \cdot 4\pi R^2$.

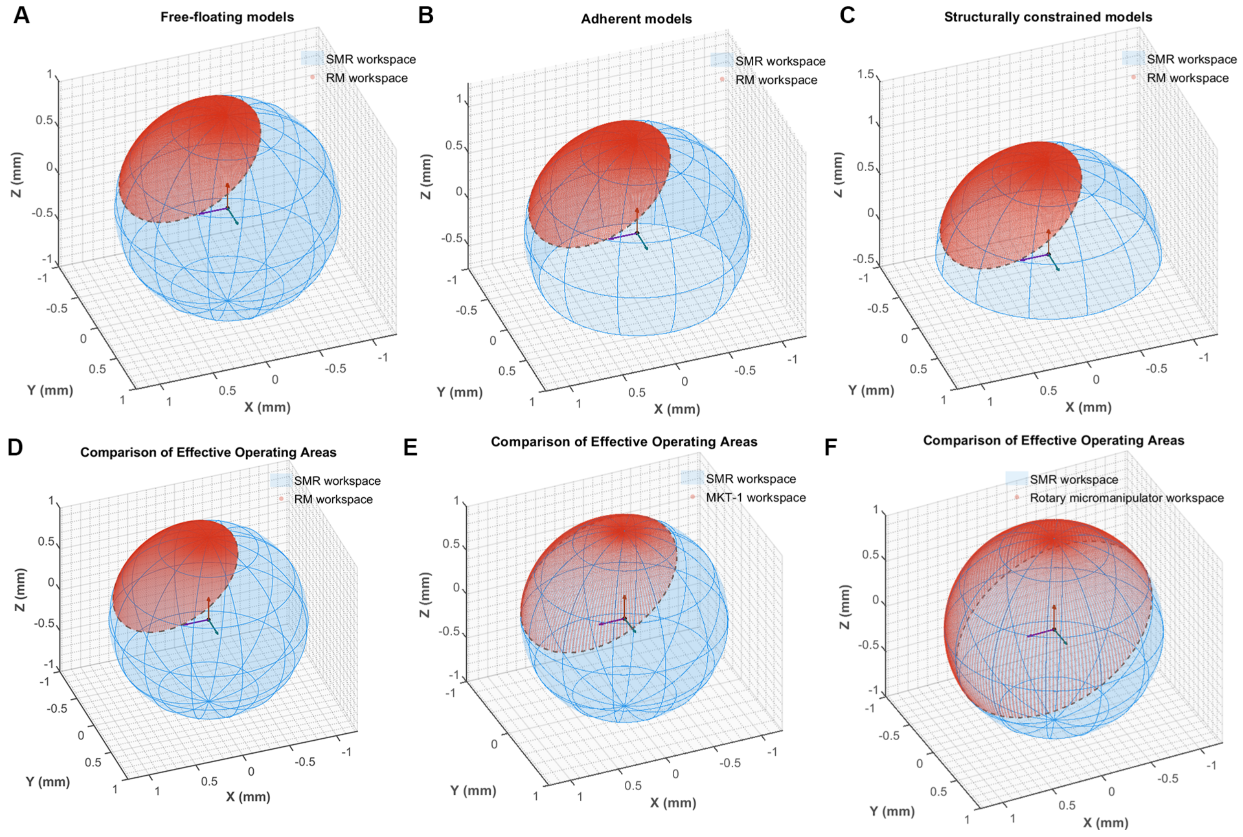

FIG. 1. Comparison of SMR with different RMs across various biological samples.

3. Structurally constrained models (e.g., samples in micro-wells, biochip devices): Samples are in highly confined environments, where access may be limited to a hemisphere or less. We conservatively model this as a maximum accessible area of $2\pi R^2$ (a hemisphere).

2. Workspace calculation for rigid manipulators (RMs)

The critical geometric constraint for a rigid manipulator (RM) is the effective operating angle β. This is defined as the maximum allowable angle between the axis of the manipulator's end-effector (e.g., micropipette) and the tangent plane at the point of contact with the sample sphere. A typical value assumed in our analysis and based on experimental practice is $\beta = 45°$. This angle fundamentally limits the accessible surface area for any RM with only translational DOF.

Standard rigid manipulators (translational DOF only): For a manipulator limited to linear translation, the accessible region from a single approach direction is a spherical cap. The half-angle of this cap, measured from the polar axis, is equal to the complement of β,

TABLE II. Comparison of different advanced manipulators

**Feature**	**DOF**	**Adjustable angle range**	**Effective operational range**	**Resolution**	**Max. Speed**
SMR this work	5	± 180°	$4\pi S^2$	0.1 μm	1.7 mm/s
MP-285 Sutter	4	None	$2\pi S^2(1-\sin\beta)$	0.04 μm	2.9 mm/s
InjectMan 4 Eppendorf	4	None	$2\pi S^2(1-\sin\beta)$	0.02 μm	10 mm/s
MX-7800 Siskiyou	4	None	$2\pi S^2(1-\sin\beta)$	0.1 μm	1.7 mm/s
m-500 RWD	4	None	$2\pi S^2(1-\sin\beta)$	0.13 μm	2.9 mm/s
MKT-1 Narishige	5	15°– 40°	$2\pi S^2[1-\sin(\beta-\delta)]$	$\sim$ 0.2 μm	$\sim$ 1 mm/s

i.e., $(90° - \beta)$. This is because the manipulator axis must be within β of the tangent plane, which is equivalent to being within $(90° - \beta)$ of the surface normal. The area of a spherical cap with half-angle θ is $2\pi S^2(1-\cos\theta)$. Therefore, the area of one inaccessible polar cap is $2\pi S^2(1-\cos(90°-\beta)) = 2\pi S^2(1-\sin\beta)$. This is the expression for models from Sutter, Eppendorf, Siskiyou, and RWD listed in Table II.

Rigid manipulators with co-axial rotation (e.g., Narishige MKT-1): Some RMs incorporate a co-axial rotational DOF (adjustable angle δ). This allows the manipulator to sweep through an arc, effectively reducing the constraint angle from β to $(\beta-\delta)$. The total accessible area for such a device is derived by substituting the new effective angle into the standard formula: $2\pi S^2[1-\sin(\beta-\delta)]$ where δ is the additional rotational angle (Figure 1E). For our calculation, we used $\delta = 12.5°$ (the midpoint of its 15°-40° range).

Workspace calculation for the SMR

Our SMR possesses five DOFs, including omnidirectional bending and continuous rotation. This allows the manipulator to orient its end-effector to achieve a perpendicular approach vector from nearly any direction relative to the sample. Therefore, its accessible workspace is not inherently limited by the angle β and is defined solely by the external environmental constraints:

1. Free-floating: $A_{\mathrm{SMR}} = 4\pi R^2$

2. Adherent: $A_{\mathrm{SMR}} \approx \frac{2}{3} \cdot 4\pi R^2$

3. Structurally constrained: $A_{\mathrm{SMR}} = 2\pi R^2$

These analyses indicate that the SMR provides a 3.4- to 6.8-fold increase in operable space compared to rigid manipulators, depending on the degree of confinement in different biological scenarios.

Supporting Note 3: 3D circular biochip

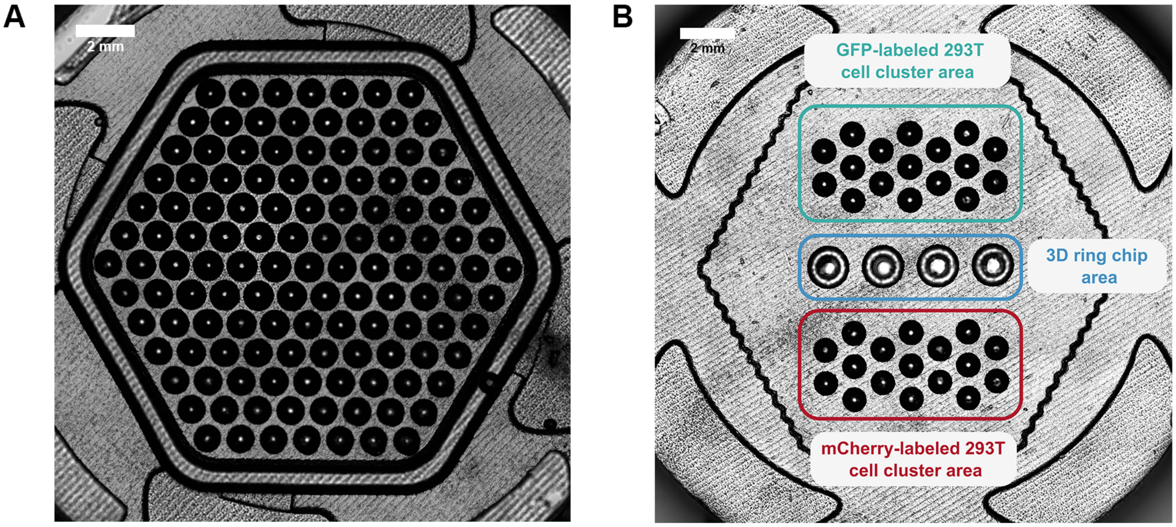

FIG. 2. Composite fluorescence image of the biochip platform showing three functional zones: GFP-labeled 293T cell clusters in the upper cultivation region (green), the central ring-shaped microarray for programmed assembly (blue), and mCherry-labeled 293T cell clusters in the lower cultivation region (red). Scale bars: 2 mm.

The biochip architecture features three distinct yet interconnected functional zones designed for programmable cell cluster manipulation. In the upper section, GFP-labeled 293T cell clusters (green fluorescence) are cultured under optimized conditions to maintain viability and fluorescence intensity. The central ring-shaped microarray serves as the active assembly area where cell clusters are precisely positioned to form predefined architectures. The lower zone contains mCherry-labeled 293T cell clusters (red fluorescence) cultivated under identical parameters to ensure experimental consistency. This tripartite design enables simultaneous monitoring of source cell viability (upper/lower zones) and engineered structure formation (central zone), while the 1.5 mm spacing between regions prevents unintended cross-contamination during robotic manipulation. Fluorescence preservation was confirmed throughout all experimental procedures.

Supporting Note 4: Limitations of Rigid Micromanipulation Systems in Cellular Ring Formation

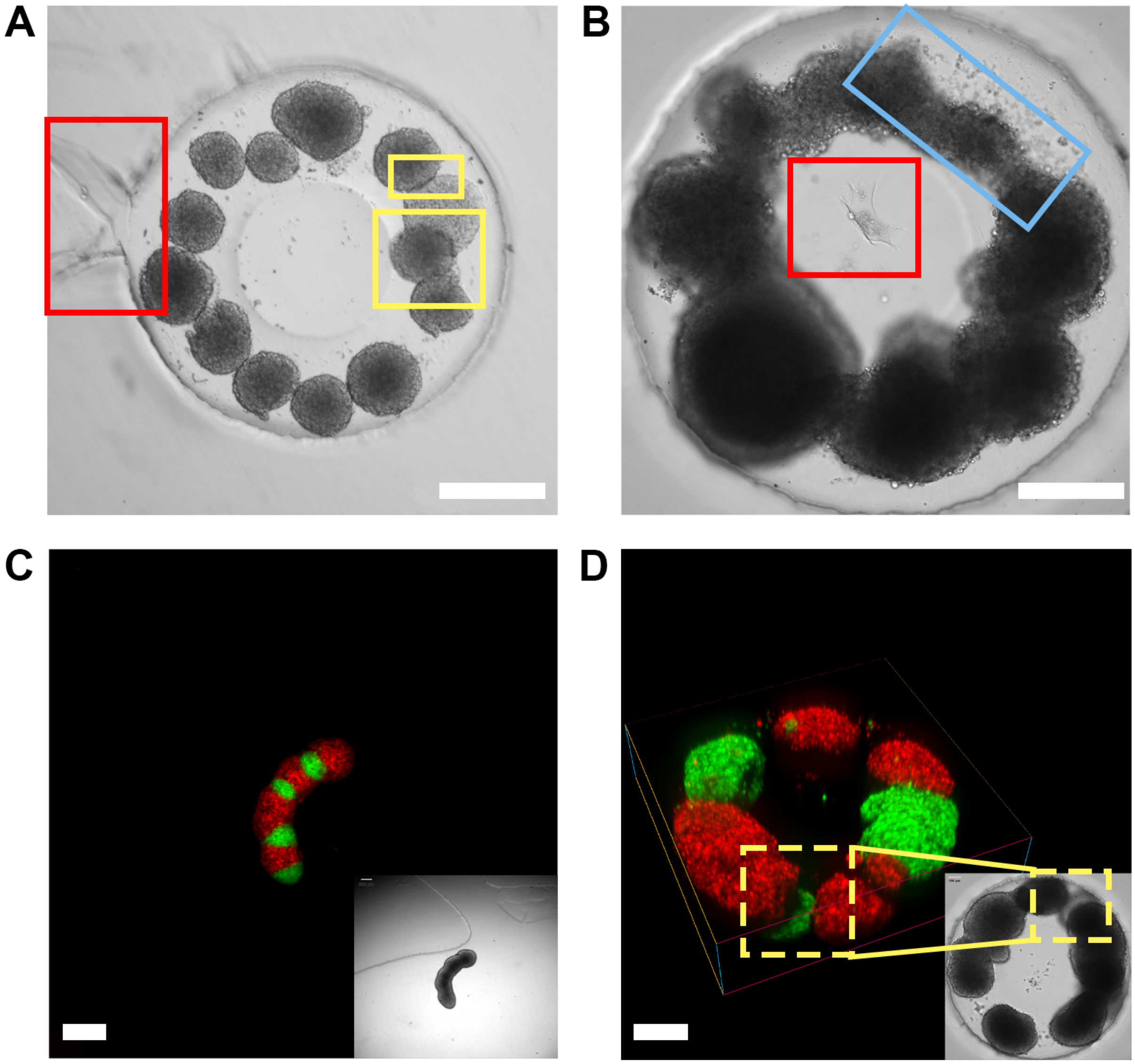

FIG. 3. Rigid micromanipulation disrupts the biochip substrate in ring-forming experiments, cell cluster overlap, damaged cell clusters, and cell cluster escape. Scale bars: 300 μm.

Conventional rigid micromanipulation instruments suffer from critical limitations in dynamic control, particularly in adjusting the microneedle's operational angle in real time. This inflexibility often leads to unintended collisions between the needle tip and delicate chip structures, causing irreversible substrate damage—as evidenced by the fractured regions highlighted in red squares (Figure 3A-B).

The operational paradigm of rigid systems relies entirely on passive placement strategies, where cell masses are positioned above target locations and left to sediment through natural gravitational settling. This approach proves fundamentally flawed in practice, as the uncontrolled settling process often leads to misaligned cellular clusters that either overlap or fail to reach their intended positions, preventing proper intercellular contact and subsequent adhesion - issues highlighted in the yellow squares of Figure 3. The fragility of this pre-adhesion state renders the system exceptionally vulnerable to even minor disturbances; routine experimental procedures such as petri dish transfer can displace unsettled cellular masses or disrupt partially formed cellular connections, as shown in Figure 3C.

These compounding limitations frequently culminate in failed ring formation attempts. While persistent manual intervention through repeated aspiration and placement cycles may eventually yield a cellular ring structure, the cumulative mechanical stress during multiple grasping operations often compromises cellular integrity, resulting in damaged and functionally impaired final architectures. The blue suqare in Figure 3B shows an incomplete cell mass in the cell ring under rigid micromanipulation.

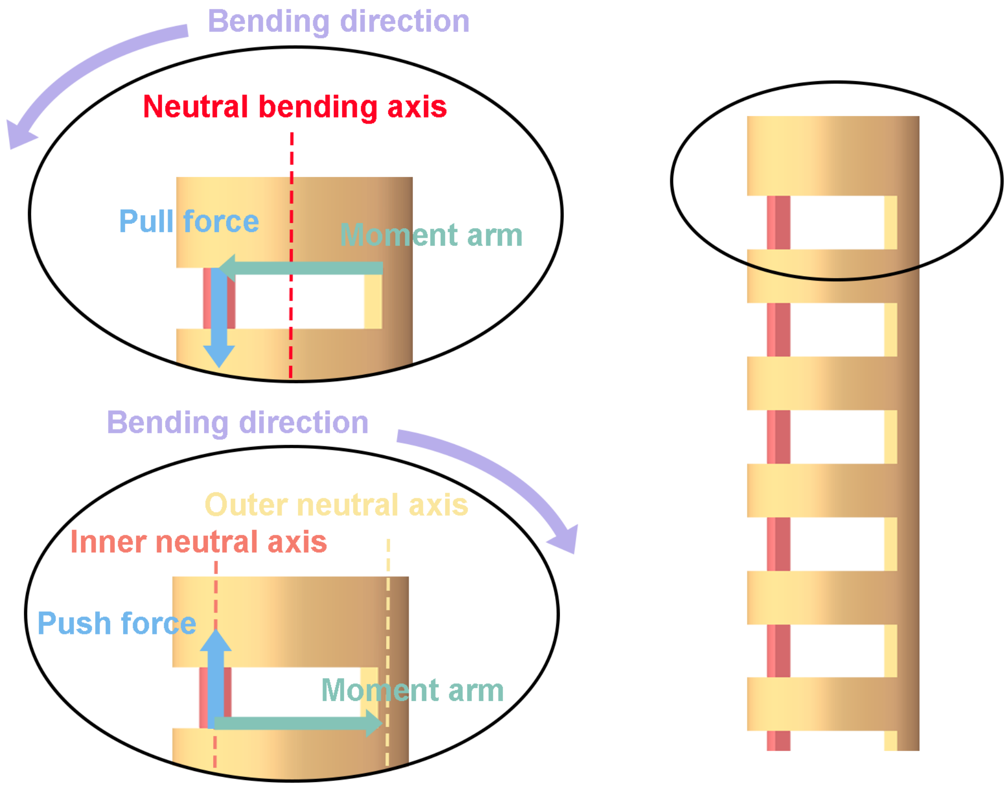

FIG. 4. Schematic representation of the bending mechanism on both sides of a soft micromanipulation robot.

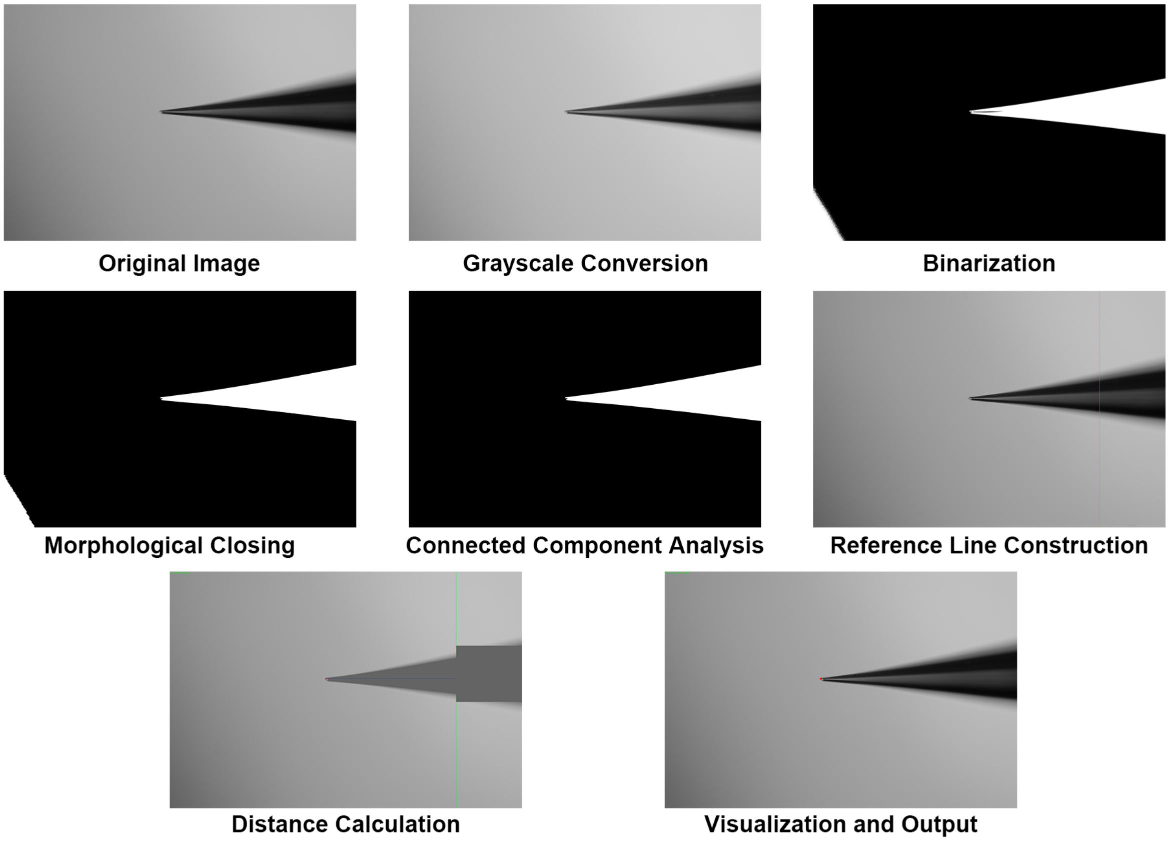

FIG. 5. The needle tip detection process begins by converting the input image to grayscale, followed by inverse binary thresholding to isolate the needle as the foreground. Noise reduction is achieved through median filtering and morphological closing to enhance structural continuity. The largest connected component is identified as the needle body, and its pixel coordinates are extracted. A reference line perpendicular to the needle's orientation is constructed to pass through the centroid of these pixels. For each pixel on the needle, the distance perpendicular to the reference line is calculated. The pixel with maximum perpendicular distance is identified as the needle tip, leveraging the geometric principle that the tip exhibits greatest deviation from the needle's central axis. This distance-based approach provides rotational invariance while efficiently localizing the extreme point of the elongated structure.

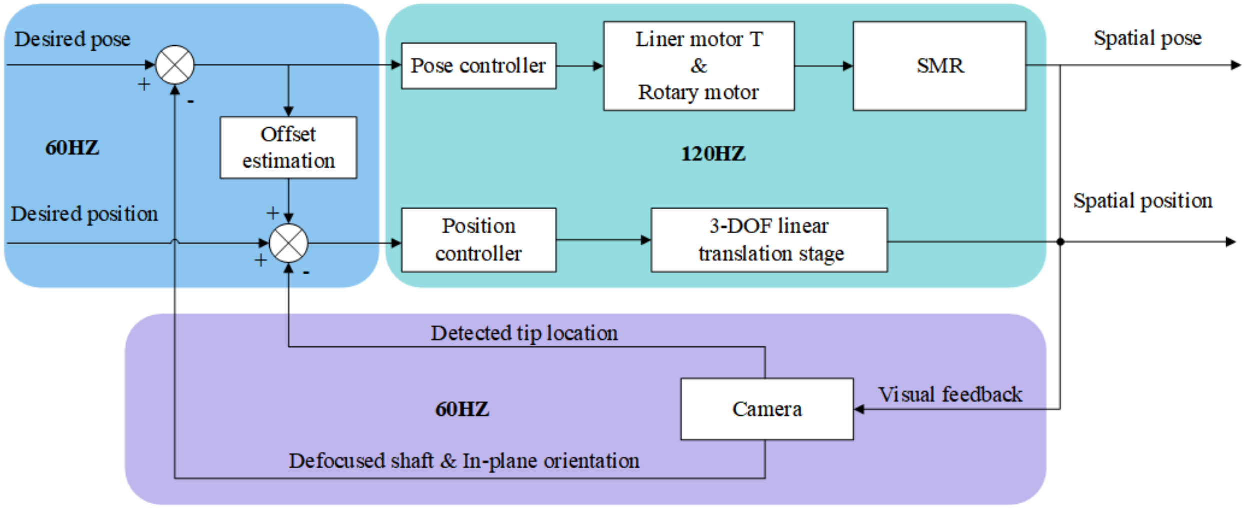

FIG. 6. SMR system control diagram.

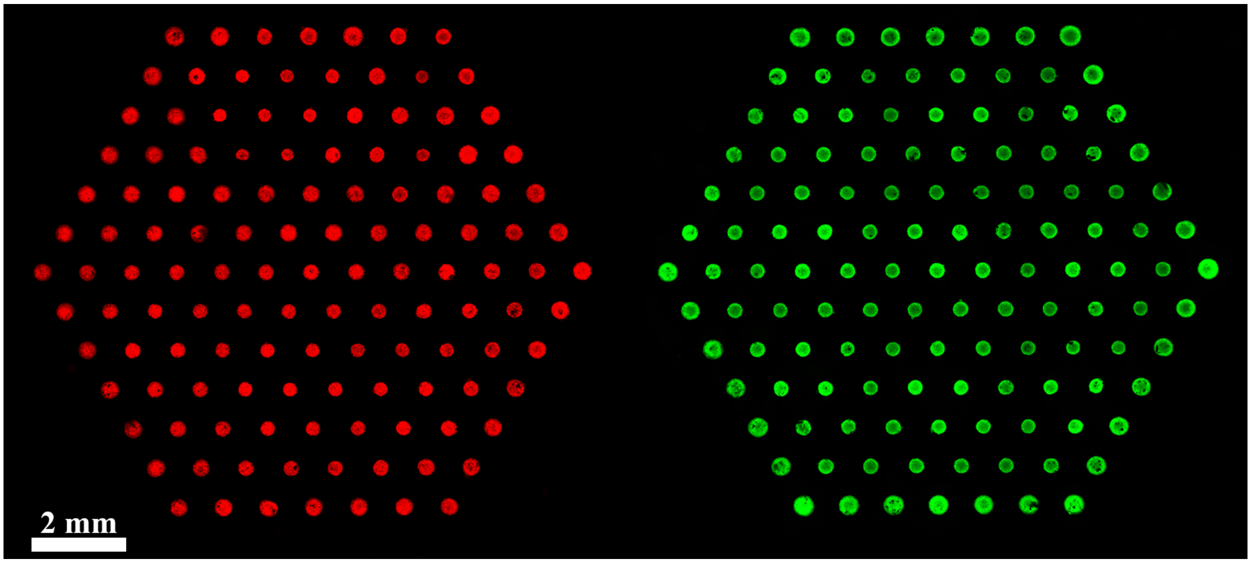

FIG. 7. 293T fluorescent cell clusters in microarray biochips on the first day of clustering.

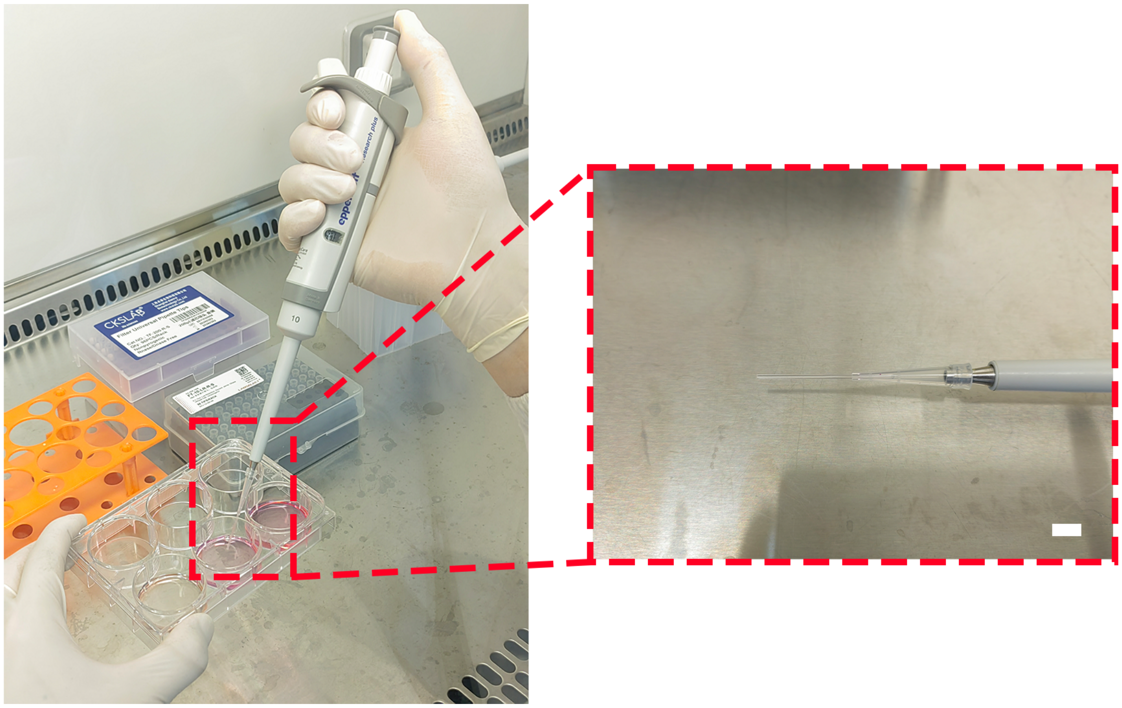

FIG. 8. A modified 10 μL volume pipette for precise manipulation of cell spheres. A 10 μL micropipette with a custom modified tip whose joints are permanently bonded to the 300 μm I.D. glass capillary tip by a light-cured acrylate-based crosslinking adhesive. This integrated tool enables experimenters to perform precise aspiration and transfer of individual 293T cell spheroids (230-250 μm diameter) under real-time microscopic visualization. Scale bar: 1 cm.

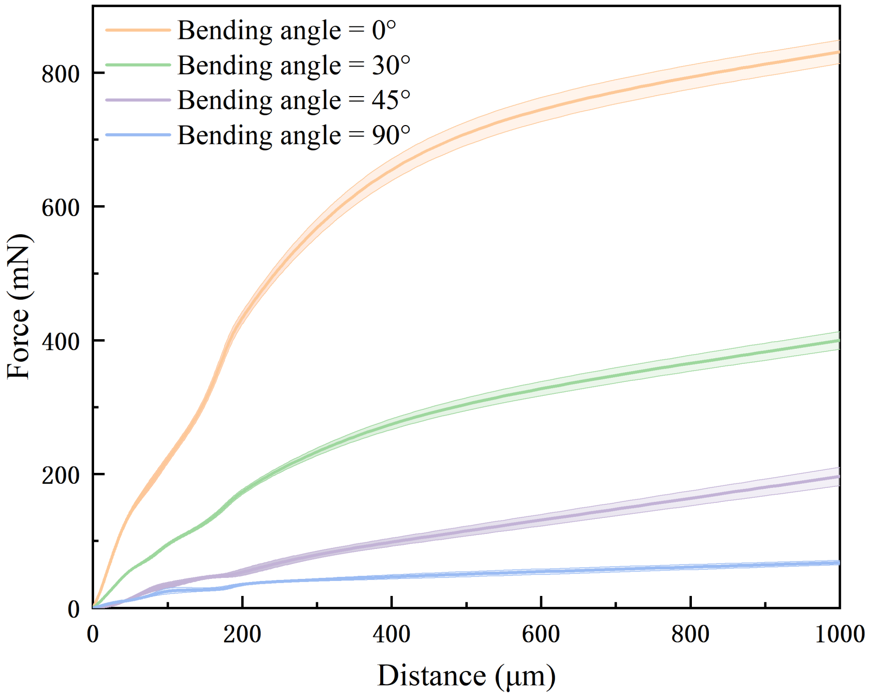

FIG. 9. Displacement-force diagram based on visual compensation displacement.
